# Management of gastric cancer peritoneal metastasis: International Gastric Cancer Association GCPM Working Group consensus statements

**DOI:** 10.1093/bjs/znag027

**Published:** 2026-03-17

**Authors:** Piers R Boshier, Daryl K A Chia, Sri G Thrumurthy, Jun Liang Teh, Maria Wobith, Maria Bencivenga, Federica Filippini, Teodora C Dumitra, Miguel Burch, Hyoung-Il Kim, Benjamin Kobitzsch, Liudmila L Kodach, Judith S E Quik, Vo Duy Long, Micha J de Neijs, Pieter C van der Sluis, Alberto M Leon-Takahashi, Yanghee Woo, Mickael Chevallay, Massimo Framarini, Paolo Morgagni, Ewelina Frejlich, Heike I Grabsch, Sheraz R Markar, Daniele Marrelli, Do Joong Park, Raghav Sundar, Zekuan Xu, Kay Khine Linn, Han Kwang Yang, Joji Kitayama, Zhenggang Zhu, Sun Young Rha, Bas Wijnhoven, Hiroharu Yamashita, Wei Peng Yong, Christelle de la Fouchardière, Magnus Nilsson, Hironori Ishigami, Johanna W Van Sandick, Florian Lordick, Brian D Badgwell, Jimmy B Y So

**Affiliations:** Department of Surgery and Cancer, Imperial College London, London, UK; Division of General Surgery (Upper Gastrointestinal Surgery), Department of Surgery, National University Hospital, Singapore; Division of General Surgery (Upper Gastrointestinal Surgery), Department of Surgery, National University Hospital, Singapore; Department of Surgery, Yong Loo Lin School of Medicine, National University of Singapore, Singapore; Division of General Surgery (Upper Gastrointestinal Surgery), Department of Surgery, National University Hospital, Singapore; Division of General Surgery (Upper Gastrointestinal Surgery), Department of Surgery, Ng Teng Fong General Hospital, Singapore; Division of General Surgery (Upper Gastrointestinal Surgery), Department of Surgery, National University Hospital, Singapore; Esophageal and Gastric Cancer Surgery Division, Department of Surgery, Dentistry, Paediatrics and Gynaecology, University of Verona, Verona, Italy; Esophageal and Gastric Cancer Surgery Division, Department of Surgery, Dentistry, Paediatrics and Gynaecology, University of Verona, Verona, Italy; Department of Surgery, McGill University, Montreal, Canada; Department of Surgery, Cedars-Sinai Medical Center, Los Angeles, California, USA; Department of Surgery, Yonsei University, Seoul, South Korea; Department of Medicine, University Cancer Centre Leipzig, Leipzig University Medical Centre, Cancer Centre Central Germany, Leipzig, Germany; Department of Pathology, The Netherlands Cancer Institute - Antoni van Leeuwenhoek Hospital, Amsterdam, The Netherlands; Department of Surgical Oncology, The Netherlands Cancer Institute - Antoni van Leeuwenhoek Hospital, Amsterdam, The Netherlands; Division of Upper GI Surgery, Department of GI Surgical, University Medical Centre, Ho Chi Minh City, Vietnam; Department of Surgery, Erasmus University Medical Centre, Rotterdam, The Netherlands; Department of Surgery, Erasmus University Medical Centre, Rotterdam, The Netherlands; Department of Surgical Oncology, National Cancer Institute, Mexico City, Mexico; Division of Surgical Oncology, Department of Surgery, City of Hope, Duarte and Orange County, California, USA; Department of Surgery, Division of Visceral Surgery, University of Geneva, Geneva, Switzerland; Department of Surgery, Morgagni-Pierantoni Hospital, Forlì, Italy; Department of Surgery, Morgagni-Pierantoni Hospital, Forlì, Italy; Department of Surgical Oncology, Faculty of Medicine, Wroclaw Medical University, Wroclaw, Poland; Division of Pathology and Data Analytics, Leeds Institute of Medical Research at St James’s, University of Leeds, Leeds, UK; Department of Pathology, GROW Research Institute for Oncology and Reproduction, Maastricht University Medical Center+, Maastricht, The Netherlands; Nuffield Department of Surgical Sciences, University of Oxford, Oxford, UK; Department of Medicine, Surgery, and Neurosciences, University of Siena, Siena, Italy; Department of Internal Medicine, Seoul National University Hospital, Seoul, South Korea; Department of Internal Medicine, Section of Medical Oncology, Yale School of Medicine, New Haven, Connecticut, USA; Department of Surgery, Division of Gastric Surgery, The First Affiliated Hospital of Nanjing Medical University, Nanjing, China; Department of Surgery, Yong Loo Lin School of Medicine, National University of Singapore, Singapore; National Cancer Center, Goyang, Republic of Korea; Department of Surgical Oncology, Japan Institute for Health Security, Tokyo, Japan; Department of Gastrointestinal Surgery, Rui Jin Hospital, Shanghai Jiao Tong University School of Medicine, Shanghai, China; Department of Internal Medicine, Yonsei University College of Medicine, Songdang Institute for Cancer Research, Yonsei University Health System, Seoul, Korea; Department of Surgery, Erasmus University Medical Centre, Rotterdam, The Netherlands; Department of Surgery, Division of Gastroenterological, General, and Transplant Surgery, Jichi Medical University, Shimotsuke, Japan; Department of Haematology-Oncology, National University Hospital, National University Cancer Institute, Singapore; Department of Medical Oncology, Institut Paoli-Calmettes, Marseille, France; Department of Upper Abdominal Diseases, Karolinska University Hospital, Stockholm, Sweden; Department of Clinical Science, Intervention, and Technology, Karolinska Institutet, Stockholm, Sweden; Department of Chemotherapy, The University of Tokyo, Tokyo, Japan; Department of Surgical Oncology, The Netherlands Cancer Institute - Antoni van Leeuwenhoek Hospital, Amsterdam, The Netherlands; Department of Medicine, University Cancer Centre Leipzig, Leipzig University Medical Centre, Cancer Centre Central Germany, Leipzig, Germany; Department of Surgical Oncology, Division of Surgery, The University of Texas MD Anderson Cancer Center, Houston, Texas, USA; Division of General Surgery (Upper Gastrointestinal Surgery), Department of Surgery, National University Hospital, Singapore; Department of Surgery, Yong Loo Lin School of Medicine, National University of Singapore, Singapore; Division of Surgical Oncology, National University Cancer Institute, Singapore

## Abstract

**Background:**

Gastric cancer peritoneal metastasis (GCPM) is a common manifestation of advanced gastric cancer, associated with poor prognosis.

**Methods:**

The International Gastric Cancer Association (IGCA) convened a multidisciplinary working group of 42 global experts from 15 countries to develop a total of 13 consensus statements addressing diagnosis, treatment, and research priorities for GCPM. Using ACcurate COnsensus Reporting Document (ACCORD)-compliant methodology, the group conducted systematic literature searches and applied a structured Delphi process with anonymous Likert-scale voting and a ≥70% consensus threshold to generate and refine consensus statements.

**Results:**

Consensus was achieved for all 13 statements among the working group during the first Delphi round, with 75–100% of respondents selecting either ‘strongly agree’ or ‘agree’. Coefficient of variation values were ≤0.23. Polling of a broader group of experts (n = 66), which included members of the working group (n = 21), during a GCPM consensus session at the 16th International Gastric Cancer Congress (IGCC) in 2025 demonstrated agreement for 12 of the 13 statements. This broader group of experts, which had greater representation from medical oncologists, did not reach consensus (52% agreement) on best practice for systemic treatment of patients with GCPM, possibly due to the rapidly evolving developments in this field of metastatic gastric cancer.

**Conclusion:**

This consensus exercise provides a foundation for globally relevant GCPM management strategies and highlights critical research needed to address significant evidence gaps that will improve patient outcomes.

## Teaser

Gastric cancer peritoneal metastasis (GCPM) remains a challenging clinical entity, characterized by poor prognosis and limited therapeutic options. This expert consensus commissioned by the International Gastric Cancer Association represents a crucial advancement of the global collective understanding of GCPM diagnosis, treatment, and research priorities.

## Introduction

Worldwide, 1.16 million people are diagnosed with gastric cancer annually and there are 0.83 million deaths from this disease^1,2^. Poor survival associated with gastric cancer primarily reflects a delay in diagnosis, with many patients presenting with advanced disease. For patients with advanced gastric cancer, the peritoneum represents the most common site of distant metastasis^3,4^.

Macroscopic peritoneal metastasis is present in around one in five patients diagnosed with gastric cancer and such patients have a median survival of 3–6 months if untreated^5–7^. A significant proportion of patients develop peritoneal metastasis after surgery with curative intent, with the peritoneum amongst the most common sites of disease recurrence^8,9^.

Current treatment of gastric cancer peritoneal metastasis (GCPM) with palliative systemic chemotherapy and, where indicated, targeted therapies may offer modest survival benefit^10^. Low penetration of systemic agents into the peritoneal cavity and resistant underlying tumour biology exemplify the challenge of treating GCPM. Over the past 25 years there has been a concerted international effort to improve the diagnosis and treatment of GCPM, including the use of intraperitoneal chemotherapy in various forms^11^. Despite significant interest in the field of GCPM, this remains a confusing and often controversial subject, the nuances of which are often neglected within major trials^12^.

The International Gastric Cancer Association (IGCA) GCPM Working Group was convened with the aim of reaching expert consensus on a broad range of subjects related to GCPM, as well as to promote future GCPM research and development of treatment guidelines. The results of a consensus exercise undertaken by the IGCA GCPM Working Group addressing diagnosis, treatment, and research priorities are reported herein.

## Methods

The methodology is designed in accordance with ACcurate COnsensus Reporting Document (ACCORD) guidelines^13^.

### Registration

The study protocol was not prospectively registered.

### Working group members

Expert stakeholders were invited to contribute to the working group. Individuals were identified or peer nominated based on their previous experience and expertise in the management of GCPM as evidenced by clinical practice and academic track record. Effort was made to ensure that the working group had broad international representation from oncology and surgical specialists. The final working group, chaired by J.B.Y.S., B.D.B., and F.L., consisted of 42 members from 15 countries. The working group had no lay members.

The working group held its first meeting on 18 June 2024. At this meeting, members agreed upon the group’s terms of reference and principal objectives, which included the creation of GCPM consensus statements. Unless otherwise stated, all meetings were held online using the Zoom platform (Zoom Communications, San Jose, CA, USA).

### Creation of consensus questions

Five members of the working group (J.B.Y.S., B.D.B., F.L., P.R.B., and D.K.A.C.) drafted an inclusive list of questions within three subject areas: diagnosis and staging; treatment; and research/future perspective.

These were subsequently presented to the full working group at a second meeting on the 9 September 2024. At this meeting, proposed questions were discussed, refined, and ratified. The final 13 questions are presented in *Table 1*.

**Table 1:** Consensus questions

Subject area	ID	Question
Diagnosis and staging	1.1	What factors predict risk of GCPM?
	1.2	By which method(s) should GCPM be diagnosed? What is the sensitivity and specificity (or other measure of diagnostic accuracy) of the following methods of diagnosing GCPM?* Laparoscopic visualization alone Laparoscopic biopsy Transcutaneous biopsy Peritoneal cytology (includes ascites and peritoneal washings)† CT PET (includes FDG, FAPI, or alternative tracer) MRI Ultrasonography Other
	1.3	By what method is GCPM best classified? Peritoneal cancer index JGCA classification Other
	1.4	What is the definition of limited GCPM? This refers to the fact that a consequence of limited GCPM is the opportunity for treatment with curative intent.
Treatment	2.1	Is systemic chemotherapy when given alone (without intraperitoneal chemotherapy) an effective treatment for GCPM?‡§ What is the expected response rate? What is the recommended systemic chemotherapy regimen?¶ Is there a role for targeted therapies/immunotherapies?
	2.2	Is intraperitoneal chemotherapy an effective treatment for GCPM?# What is the expected response rate? What intraperitoneal chemotherapy agents are recommended?¶ Does the mode of intraperitoneal chemotherapy administration influence efficacy?
	2.3	What factors predict efficacy of intraperitoneal chemotherapy?
	2.4	How should GCPM therapeutic response be evaluated? Disease-specific metrics and outcomes Patient-reported outcomes
	2.5	Is there a role for conversion surgery in the treatment of GCPM?#** What is the definition of conversion surgery? What should the objective(s) of conversion surgery be? By what criteria should patients be selected for conversion surgery? Does HIPC administered at the time of surgery improve outcomes?
	2.6	What should the objectives of palliative care in GCPM be?††
	2.7	What evidence is there for prophylactic treatment of GCPM?
Research/future perspective	3.1	What novel approaches are there for detecting, staging, and/or monitoring GCPM?
	3.2	What potential novel therapeutic strategies are there for treating GCPM?

*Peritoneal metastasis in this context refers to the presence of macroscopic peritoneal metastasis. †Positive peritoneal cytology without macroscopic peritoneal metastasis is not considered in this consensus. ‡Efficacy refers specifically to the treatment of peritoneal metastasis as opposed to the primary tumour. §In the context of this consensus, treatment efficacy may be defined by: overall survival, progression free survival, regression of peritoneal metastasis, and/or symptom control. ¶Please consider not only the type of agents used but also the duration of treatment. #It is assumed that systemic chemotherapy will be given before, after, or during (as bidirectional therapy) intraperitoneal chemotherapy administration. **In this context, conversion surgery may include aspects of cytoreduction. Please consider this when answering points 1 and 2. ††Please consider factors that may be specific to GCPM and its sequelae. GCPM, gastric cancer peritoneal metastasis; FDG, fluorodeoxyglucose; FAPI, fibroblast activation protein inhibitor; JGCA, Japanese Gastric Cancer Association; HIPC, hyperthermic intraperitoneal chemotherapy.

### Review of existing scientific evidence

Questions were allocated to working group members who agreed to review and summarize existing scientific evidence that would inform the creation of consensus statements. Contributing members were requested to perform a systematic literature search using online databases (for example Ovid and MEDLINE) and relevant search terms with stated inclusion/exclusion criteria. Where considered relevant, review of specific journals (for example *Gastric Cancer* and *Pleura and Peritoneum*) and trial registries (for example ClinicalTrials.gov) was performed. Evidence was then summarized and presented in a concise (1–2 page) format using a standard template (*Supplementary material*).

Finally, contributing members were asked to create draft consensus statements and grade the level of supporting evidence using the Grading of Recommendations Assessment, Development, and Evaluation (GRADE) classification system^14^.

### Presentation of evidence and proposed consensus statements

Evidence and proposed consensus statements were presented to working group members during two meetings on the 19 November 2024 and 13 January 2025.

### Delphi panel and consensus process

Consensus of opinion regarding each of the 13 statements was evaluated using Delphi methodology^15^. The Delphi panel was made up of all members of the IGCA GCPM Working Group. There were no declared or perceived conflicts of interests among working group members that would preclude their participation in the Delphi process. All supporting evidence, consensus statements, voting, and discussions were written/conducted in English.

Anonymous voting was conducted using the Poll Everywhere (San Francisco, CA, USA) platform. Invitations to participate were sent via e-mail and were only re-sent once if there was no response. Responses for each consensus statement were acquired using a five-point Likert-scale scoring system: strongly agree (5); agree (4); neutral (3); disagree (2); and strongly disagree (1).

In line with previous Delphi studies, the threshold for consensus was defined as ≥70% of responding panellists indicating that they either ‘strongly agree’ or ‘agree’ with the proposed statement. Using the numerical values assigned to the Likert scale, the coefficient of variation (CV) of responses (standard deviation divided by the mean) was calculated to assess the degree of dispersion.

During each round, panel members were encouraged to comment on each consensus statement and to suggest modifications and/or additional statements. Where indicated, consensus statements were modified before subsequent voting rounds. The Delphi process was closed when all statements reached either consensus or agreed non-consensus.

After the first round of Delphi voting, a planned in-person GCPM consensus session was held at the 16th International Gastric Cancer Congress (IGCC) in Amsterdam (9 May 2025). Over 100 people attended this session that was open to all members of the IGCA GCPM Working Group and congress attendees. Discussion of each statement by session participants, including both surgeons and medical oncologists, was led by an invited expert panel made up of three IGCA GCPM Working Group members (J.B.Y.S., C.d.l.F., and B.D.B.). This session was used as an opportunity to present each statement (±revisions) after the first round of Delphi voting and to conduct further consensus voting amongst members of the IGCA GCPM Working Group, as well as a wider group of experts to establish broader acceptance of the proposed statements.

### Ethics

Formal ethical approval was not required to complete this consensus exercise.

## Results

A summary of the supporting evidence and accompanying references for each consensus statement is provided in the *Supplementary material*. Statements submitted for first- and second-round voting, including proposed revisions, are presented in *Table S1*.

The first-round Delphi consensus voting was undertaken from 30 April to 6 May 2025. In total, 28 members (67%) of the working group voted in this round. A summary of the results of first-round voting is presented in *Fig. S1*. All statements reached consensus, with 75–100% of respondents selecting either ‘strongly agree’ or ‘agree’. CV values for all 13 statements were ≤0.23. Anonymous feedback provided by first-round respondents was reviewed and statements were amended accordingly. Details of amended statements after the first round of voting are presented in the *Supplementary material*.

**Fig. 1:**
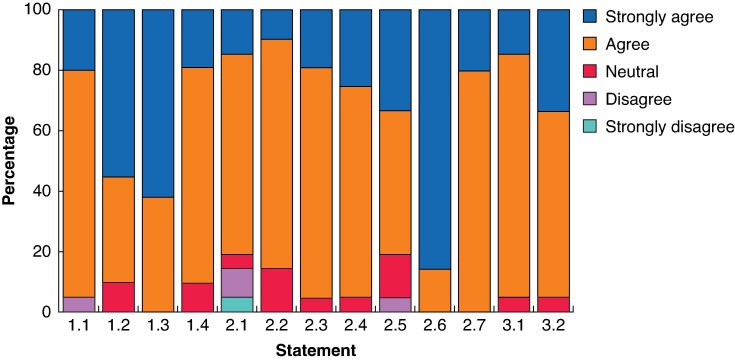
Results of voting for the second Delphi round amongst IGCA GCPM Working Group members IGCA, International Gastric Cancer Association; CGPM, gastric cancer peritoneal metastasis.

**Fig. 2:**
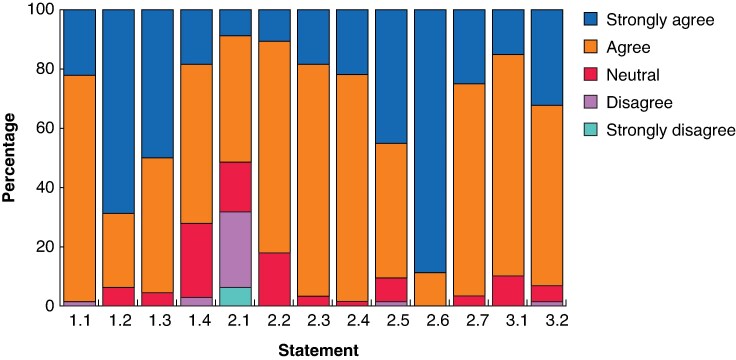
Results of open voting during the GCPM consensus session at the 16th IGCC in 2025 CGPM, gastric cancer peritoneal metastasis; IGCC, International Gastric Cancer Congress.

During the GCPM consensus session at the 16th IGCC in 2025, a broader group of experts, including surgeons, oncologists, pathologists, oncology nurses, and a patient representative, participated. In total, 66 individuals voted on each statement, including 21 members (50%) of the IGCA GCPM Working Group who were present in person during the session. Final consensus statements, as well as voting results, are summarized in *Table 2*, *Fig. 1*, and *Fig. 2*. Amongst members of the IGCA GCPM Working Group, all amended statements again reached consensus. For three statements (1.2, 2.2, and 2.5), overall agreement was lower in the second round of voting compared with the first round of voting. The difference was greatest for statement 2.2, where agreement (‘strongly agree’ or ‘agree’) decreased from 100% to 86%.

**Table 2:** Final consensus statements and voting results

ID	Consensus statements	Round 1, working group (*n* = 28)		GCPM consensus session at the 16th IGCC in 2025			
				Working group (*n* = 21)		All (*n* = 66)	
		Agreement (%)	CV	Agreement (%)	CV	Agreement (%)	CV
1.1	Multiple patient-specific (female sex, younger age, and ethnicity) and tumour-specific (T4a, cN2+, positive peritoneal cytology, diffuse type, presence of SRC, genomically stable (TCGA), EMT subtype (ACRG), CLDN18.2-ARHGAP overexpression, and mutations in CDH-1, CTNNA 1, and RHOA genes) risk factors for peritoneal carcinomatosis have been acknowledged. Level of evidence: moderate/very high	83	0.21	95	0.16	98	0.12
1.2	CT is a suitable non-invasive and accessible first-line investigation for GCPM. Where possible and appropriate (for example in patients with potentially resectable disease), staging laparoscopy with peritoneal cytology and histopathology of peritoneal deposits remains the ‘gold standard’ for diagnosing GCPM. Level of evidence: low/very low	96	0.13	90	0.15	94	0.13
1.3	For patients who are being considered for multimodal treatment of GCPM, quantitative classification of peritoneal metastasis, using either the PCI (Sugarbaker) or P1a/b/c (Japanese) classification system, is crucial for assessing disease extent and predicting the feasibility of complete cytoreduction. Level of evidence: low	97	0.12	100	0.11	95	0.13
1.4	The definition of limited peritoneal metastases could be PCI ≤6 (Sugarbaker classification) or P1a (Japanese classification), but specification of the definition for subgroups may help to work towards more patient-tailored treatment. Level of evidence: low	89	0.18	90	0.13	72	0.19
2.1	Optimal drug regimens and duration of treatment in GCPM have not been defined. In addition to best supportive care alone, systemic chemotherapy, often a combination of platinum compounds, fluoropyrimidines, and taxanes, remains the standard of care for patients with GCPM. Use of targeted therapies and immunotherapies should be individualized to patients. Level of evidence: high/low	75	0.23	81	0.26	52	0.35
2.2	Intraperitoneal chemotherapy administered as HIPEC or PIPAC is still investigational. While normothermic intraperitoneal paclitaxel can be recommended in clinical practice in Eastern Asian populations, Western trials are still needed for the sake of generalizability. Level of evidence: HIPEC: low; PIPAC: low; normothermic intraperitoneal paclitaxel: moderate to high	100	0.09	86	0.13	82	0.14
2.3	Multiple factors, including patient performance status, presence of malignant ascites, peritoneal disease burden, tumour chemosensitivity, treatment regimen, and mode of delivery, may predict efficacy of intraperitoneal chemotherapy. Level of evidence: low	89	0.14	95	0.12	97	0.11
2.4	Survival and radiological response remain the most widely used metrics of therapeutic response in patients with GCPM. In cases where conversion surgery is being considered, further evaluation by laparoscopy and the PRGS should be undertaken, allowing for more accurate evaluation of GCPM burden. Greater emphasis on patient-reported outcomes is also necessary. Level of evidence: low/very low	85	0.19	95	0.12	98	0.11
2.5	Conversion surgery may be a therapeutic option for selected patients with limited GCPM. The impact of adding HIPEC at the time of conversion surgery warrants further investigation. Level of evidence: low	82	0.22	81	0.20	90	0.16
2.6	Palliative care should provide the best symptom management possible for the patient and their family/caregivers, with the objective of providing optimal quality of life in the context of their goals of care. Level of evidence: low	96	0.11	100	0.07	100	0.07
2.7	While prophylactic intraperitoneal treatments show promise in reducing peritoneal metastasis and improving survival outcomes in high-risk gastric cancer populations, this application is still experimental and further clinical trials are needed to establish the efficacy across diverse populations and the impact on survival. Level of evidence: low/very low	86	0.18	100	0.10	96	0.12
3.1	FAPI PET and DW MRI are emerging imaging modalities for the assessment of GCPM. Additionally, confirmatory testing of peritoneal fluid for genetic and protein biomarkers offers a feasible and minimally invasive strategy to evaluate the progression or regression of GCPM. Level of evidence: low/very low	82	0.15	95	0.11	90	0.12
3.2	Novel therapeutic strategies for GCPM should seek to leverage the unique anatomy and tumour microenvironment of the peritoneum, including the development of enhanced regional and targeted therapies. Level of evidence: very low	83	0.17	95	0.13	93	0.15

GCPM, gastric cancer peritoneal metastasis; IGCC, International Gastric Cancer Congress; CV, coefficient of variation; SRC, signet ring carcinoma; EMT, epithelial-to-mesenchymal transition; PCI, peritoneal carcinoma index; HIPEC, hyperthermic intraperitoneal chemotherapy; PIPAC, pressurized intraperitoneal aerosolized chemotherapy; PRGS, Peritoneal Regression Grading Score; FAPI, fibroblast activation protein inhibitor; DW, diffusion-weighted.

When voting by all attendees was considered, consensus agreement was reached for all statements, with the exception of statement 2.1 (treatment—systemic therapy; 52% ‘strongly agree’ or ‘agree’). In general, the level of consensus within this broader group of experts was equivalent to or higher than that reached by the IGCA GCPM Working Group; exceptions were statements 1.4 (diagnosis and staging—definition of limited GCPM; 72%), 2.1 (treatment—systemic chemotherapy; 52%), and 2.2 (treatment—intraperitoneal chemotherapy; 82%), which saw lower consensus amongst the broader group of experts.

## Discussion

GCPM remains a challenging clinical entity, characterized by poor prognosis and limited therapeutic options. This expert consensus by the IGCA GCPM Working Group represents a major advancement of the global collective understanding of GCPM diagnosis, treatment, and research priorities. Members of the working group reached agreement on all proposed statements across two rounds of Delphi voting. The process provided the opportunity for amendments that improved the clarity and impact of each statement. Minor variations in voting between each round were consistent with the number and clinical background of the working group members participating in voting.

A single statement did not reach consensus amongst a broader group of stakeholders. This related to current evidence for the effectiveness of systemic treatment when given alone (without intraperitoneal chemotherapy) to treat GCPM. Whilst consensus was reached on this subject amongst the working group members, the inability to reach consensus by a broader group of experts, which had greater representation from medical oncologists, potentially reflected the acknowledged complexity of this subject and significant variations in practice between individual centres and global regions.

During the Delphi process, several important challenges regarding GCPM patient management were highlighted. Diagnostic challenges remain a major barrier to caring for patients with GCPM. Staging laparoscopy was reaffirmed as the ‘gold standard’ modality to diagnose and stage GCPM due to its high specificity and capacity to directly visualize disease extent. However, limitations related to invasiveness, cost, and the diagnostic inaccuracy of peritoneal cytology emphasize the need for novel non-invasive diagnostics. Promising avenues, such as fibroblast activation protein inhibitor (FAPI) PET and molecular markers from peritoneal fluid, are recognised^16–25^, although, currently, they are not widely used. Liquid biopsy techniques may also hold promise to transform current practice, enabling timely intervention before widespread dissemination occurs.

Treatment of GCPM continues to be hindered by the biological complexity and chemoresistance of peritoneal metastases^26^. The consensus highlighted the modest benefit of systemic chemotherapy, reflecting limitations in drug delivery to the peritoneal cavity and the heterogeneous nature of gastric cancers, especially those with signet ring cell histology. Encouraging real-world data on triplet regimens, such as fluorouracil, leucovorin, oxaliplatin, and docetaxel (FLOT), provide a foundation for systemic treatment of Western patients^27^, but the lack of dedicated clinical trials for GCPM populations represents a significant knowledge gap. Furthermore, a better understanding of gastric cancer biology, together with the identification of novel tumour biomarkers and the development of targeted therapies, may open new perspectives in the management of GCPM.

Intraperitoneal chemotherapy has emerged as a focal point of ongoing research, with normothermic infusion of taxane-based chemotherapy showing the most promise among intraperitoneal methods^11,28^. Although hyperthermic intraperitoneal chemotherapy (HIPEC) has been extensively studied, definitive evidence of survival benefit remains elusive^29,30^. Pressurized intraperitoneal aerosolized chemotherapy (PIPAC), the most recent intraperitoneal chemotherapy technique, is promising theoretically, but robust clinical evidence supporting its efficacy is still lacking^11^. This ambiguity underscores the critical need for well-designed RCTs to clarify indications, optimal agents, and delivery methods.

Likewise, conversion surgery after systemic (±intraperitoneal) treatment remains a potentially valuable therapeutic option for carefully selected patients with limited peritoneal disease burdens. The evidence suggests that complete cytoreduction is key to improved survival, but uncertainty remains around criteria for patient selection, timing, and integration with intraperitoneal therapies. Given the reported heterogeneity in clinical outcomes, further prospective studies are warranted to refine patient selection for surgery and optimize multimodal treatment protocols.

Prospective clinical trials specifically designed for GCPM populations across the full range of treatment modalities are needed. Future studies should not only address systemic chemotherapy regimens but also the integration of targeted agents, immunotherapies, and novel intraperitoneal drugs and drug delivery systems. Understanding the molecular and immunological microenvironment of peritoneal metastases will be crucial in guiding personalized therapy. The role of conversion surgery, while promising, requires standardization through prospective multicentre studies that define patient selection criteria, treatment modalities, and surgical techniques. Finally, expanding research into palliative care interventions tailored to the unique challenges regarding GCPM patients is critical. Interdisciplinary trials incorporating surgical, medical, and supportive care perspectives should aim to improve symptom control, quality of life, and psychosocial outcomes.

The final working group consisted of 42 experts from 15 countries, spanning medical and surgical oncology and surgical disciplines, ensuring diverse clinical perspectives and reducing geographical bias. By establishing clear terms of reference and focusing on diagnosis, treatment, and research, the group maintained clinical relevance. Each consensus statement was underpinned by comprehensive evidence reviews and graded using the GRADE system, enhancing transparency and confidence in recommendations.

An iterative Delphi process with anonymous voting minimized bias and groupthink, encouraging independent expert judgment. The low CV values (≤0.23) across voting rounds reflect the consistency of the expert opinions; in addition, the opportunity for members to suggest statement modifications allowed for expert feedback and for statements to evolve.

Finally, the hybrid format of virtual meetings and an in-person GCPM consensus session at the 16th IGCC in 2025 promoted broad engagement. Engagement with a wider body of stakeholders, including oncology nurses, pathologists, and a patient representative, during the GCPM consensus session helped to ensure that the statements had a broader relevance.

The consensus process would have undoubtably benefited from greater representation from members of multidisciplinary teams, including medical oncologists, pathologists, and radiologists, as well as other allied healthcare professionals and patient representatives. Due to time limitations and the challenge of all working group members being present at the 16th IGCC in 2025, it was not possible to ensure that all 42 members could vote on the statements in each round. Whilst the proportion of members voting in round one and round two was 67% and 50% respectively, across both rounds, 83% of members were able to cast their vote. Broader polling of those who attended the GCPM consensus session at the 16th IGCC in 2025 is likely to be subject to selection bias.

This consensus process demonstrates a rigorous and collaborative approach, enhancing clinical understanding and fostering international coordination of care for GCPM patients. Whilst the consensus process has identified an up-to-date evidence base and broad agreement between experts on many aspects of GCPM and its management, it also highlights substantial unmet needs in the diagnosis and treatment of this condition.

The findings provide a roadmap for future research efforts aimed at improving outcomes for patients with GCPM. Continued global collaboration, alongside innovations in therapeutics and technology, will be key to addressing the complex challenges posed by GCPM.

## Supplementary Material

znag027_Supplementary_Data

## Data Availability

Data are available upon written request to the corresponding author.

## References

[1] GBD 2016 Causes of Death Collaborators . Global, regional, and national age-sex specific mortality for 264 causes of death, 1980-2016: a systematic analysis for the Global Burden of Disease Study 2016. Lancet 2017;390:1151–1210 28919116 10.1016/S0140-6736(17)32152-9 PMC5605883

[2] GBD 2016 Disease and Injury Incidence and Prevalence Collaborators . Global, regional, and national incidence, prevalence, and years lived with disability for 328 diseases and injuries for 195 countries, 1990-2016: a systematic analysis for the Global Burden of Disease Study 2016. Lancet 2017;390:1211–1259 28919117 10.1016/S0140-6736(17)32154-2 PMC5605509

[3] Sirody J, Kaji AH, Hari DM, Chen KT. Patterns of gastric cancer metastasis in the United States. Am J Surg 2022;224:445–448 35144812 10.1016/j.amjsurg.2022.01.024

[4] Wang L, Liang B, Jiang Y, Huang G, Tang A, Liu Z et al Subsite-specific metastatic organotropism and risk in gastric cancer: a population-based cohort study of the US SEER database and a Chinese single-institutional registry. Cancer Med 2023;12:19595–19606 37740601 10.1002/cam4.6583 PMC10587925

[5] Rijken A, Lurvink RJ, Luyer MDP, Nieuwenhuijzen GAP, van Erning FN, van Sandick JW et al The burden of peritoneal metastases from gastric cancer: a systematic review on the incidence, risk factors and survival. J Clin Med 2021;10:4882 34768402 10.3390/jcm10214882 PMC8584453

[6] Thomassen I, van Gestel YR, van Ramshorst B, Luyer MD, Bosscha K, Nienhuijs SW et al Peritoneal carcinomatosis of gastric origin: a population-based study on incidence, survival and risk factors. Int J Cancer 2014;134:622–628 23832847 10.1002/ijc.28373

[7] van der Sluis K, Vollebergh MA, Kodach LL, van Dieren JM, de Hingh I, Wijnhoven BPL et al The clinical implications of staging laparoscopy in the diagnostic workup of gastric cancer patients: a population based study. Eur J Surg Oncol 2024;50:108569 39134081 10.1016/j.ejso.2024.108569

[8] Noh SH, Park SR, Yang HK, Chung HC, Chung IJ, Kim SW et al Adjuvant capecitabine plus oxaliplatin for gastric cancer after D2 gastrectomy (CLASSIC): 5-year follow-up of an open-label, randomised phase 3 trial. Lancet Oncol 2014;15:1389–1396 25439693 10.1016/S1470-2045(14)70473-5

[9] Glatz T, Verst R, Kuvendjiska J, Bronsert P, Becker H, Hoeppner J et al Pattern of recurrence and patient survival after perioperative chemotherapy with 5-FU, leucovorin, oxaliplatin and docetaxel (FLOT) for locally advanced esophagogastric adenocarcinoma in patients treated outside clinical trials. J Clin Med 2020;9:2654 32824326 10.3390/jcm9082654 PMC7464040

[10] Koemans WJ, Lurvink RJ, Grootscholten C, Verhoeven RHA, de Hingh IH, van Sandick JW. Synchronous peritoneal metastases of gastric cancer origin: incidence, treatment and survival of a nationwide Dutch cohort. Gastric Cancer 2021;24:800–809 33495964 10.1007/s10120-021-01160-1

[11] Boshier PR, Tekkis N, Baggaley A, Robb HD, Lafaurie G, Simkens G et al Outcomes of intraperitoneal chemotherapy for the treatment of gastric cancer with peritoneal metastasis: a comprehensive systematic review and meta-analysis. Eur J Surg Oncol 2025;51:109499 39644811 10.1016/j.ejso.2024.109499

[12] Brandl A, van Sandick JW. Treatment of gastric cancer peritoneal metastases: role of cytoreductive surgery and hyperthermic intraperitoneal chemotherapy. Br J Surg 2024;111:znae149 38953711 10.1093/bjs/znae149 PMC11217987

[13] Gattrell WT, Logullo P, van Zuuren EJ, Price A, Hughes EL, Blazey P et al ACCORD (ACcurate COnsensus Reporting Document): a reporting guideline for consensus methods in biomedicine developed via a modified Delphi. PLoS Med 2024;21:e1004326 38261576 10.1371/journal.pmed.1004326 PMC10805282

[14] Guyatt G, Oxman AD, Akl EA, Kunz R, Vist G, Brozek J et al GRADE guidelines: 1. Introduction - GRADE evidence profiles and summary of findings tables. J Clin Epidemiol 2011;64:383–394 21195583 10.1016/j.jclinepi.2010.04.026

[15] Dalkey N, Helmer O. An experimental application of the Delphi method to the use of experts. Manag Sci 1963;9:458–467

[16] Ruan D, Zhao L, Cai J, Xu W, Sun L, Li J et al Evaluation of FAPI PET imaging in gastric cancer: a systematic review and meta-analysis. Theranostics 2023;13:4694–4710 37649615 10.7150/thno.88335 PMC10465231

[17] Pang Y, Zhao L, Luo Z, Hao B, Wu H, Lin Q et al Comparison of ^68^Ga-FAPI and ^18^F-FDG uptake in gastric, duodenal, and colorectal cancers. Radiology 2021;298:393–402 33258746 10.1148/radiol.2020203275

[18] Shimura T, Toden S, Kandimalla R, Toiyama Y, Okugawa Y, Kanda M et al Genomewide expression profiling identifies a novel miRNA-based signature for the detection of peritoneal metastasis in patients with gastric cancer. Ann Surg 2021;274:e425–e434 31663973 10.1097/SLA.0000000000003647 PMC7577555

[19] Ohzawa H, Kumagai Y, Yamaguchi H, Miyato H, Sakuma Y, Horie H et al Exosomal microRNA in peritoneal fluid as a biomarker of peritoneal metastases from gastric cancer. Ann Gastroenterol Surg 2020;4:84–93 32021962 10.1002/ags3.12296 PMC6992685

[20] van der Sluis K, van Sandick JW, Vollebergh MA, van Dieren JM, Hugen N, Hartemink KJ et al Improving diagnostic accuracy of identifying gastric cancer patients with peritoneal metastases: tumor-guided cell-free DNA analysis of peritoneal fluid. Oncogene 2024;43:1877–1882 38654110 10.1038/s41388-024-03034-z

[21] Gwee YX, Chia DKA, So J, Ceelen W, Yong WP, Tan P et al Integration of genomic biology into therapeutic strategies of gastric cancer peritoneal metastasis. J Clin Oncol 2022;40:2830 35649219 10.1200/JCO.21.02745 PMC9390822

[22] Allan Z, Witts S, Wong DJ, Lee MM, Tie J, Tebbutt NC et al Peritoneal tumor DNA as a prognostic biomarker in gastric cancer: a systematic review and meta-analysis. JCO Precis Oncol 2024;8:e2300546 38513167 10.1200/PO.23.00546

[23] Jin J, Son M, Kim H, Kim H, Kong SH, Kim HK et al Comparative proteomic analysis of human malignant ascitic fluids for the development of gastric cancer biomarkers. Clin Biochem 2018;56:55–61 29654727 10.1016/j.clinbiochem.2018.04.003

[24] Ueda A, Yuki S, Ando T, Hosokawa A, Nakada N, Kito Y et al CA125 kinetics as a potential biomarker for peritoneal metastasis progression following taxane-plus-ramucirumab administration in patients with advanced gastric cancer. Cancers (Basel) 2024;16:871 38473233 10.3390/cancers16050871 PMC10930593

[25] Emoto S, Ishigami H, Yamashita H, Yamaguchi H, Kaisaki S, Kitayama J. Clinical significance of CA125 and CA72-4 in gastric cancer with peritoneal dissemination. Gastric Cancer 2012;15:154–161 21892754 10.1007/s10120-011-0091-8

[26] Wang R, Song S, Harada K, Ghazanfari Amlashi F, Badgwell B, Pizzi MP et al Multiplex profiling of peritoneal metastases from gastric adenocarcinoma identified novel targets and molecular subtypes that predict treatment response. Gut 2020;69:18–31 31171626 10.1136/gutjnl-2018-318070 PMC6943252

[27] Guchelaar NAD, Noordman BJ, Welten MW, van Santen MT, de Neijs MJ, Koolen SLW et al Systemic treatment strategies and outcomes of patients with synchronous peritoneal metastases of gastric origin: a nationwide population-based study. J Natl Compr Canc Netw 2024;22:405–412 39074509 10.6004/jnccn.2024.7013

[28] Yan C, Yang Z, Shi Z, Lu S, Shi M, Nie M et al Intraperitoneal and intravenous paclitaxel plus S-1 versus intravenous paclitaxel plus S-1 in gastric cancer patients with peritoneal metastasis: results from the multicenter, randomized, phase 3 DRAGON-01 trial. J Clin Oncol 2025; 43(Suppl): 327

[29] Rau B, Lang H, Koenigsrainer A, Gockel I, Rau HG, Seeliger H et al Effect of hyperthermic intraperitoneal chemotherapy on cytoreductive surgery in gastric cancer with synchronous peritoneal metastases: the phase III GASTRIPEC-I trial. J Clin Oncol 2024;42:146–156 37906724 10.1200/JCO.22.02867 PMC10824373

[30] Quik JSE, Van der Sluis K, van der Noort V, Retel V, Luyer MDP, van Hellemond IEG et al 2096MO Systemic therapy, gastrectomy and CRS/HIPEC vs systemic therapy alone for gastric cancer with limited peritoneal dissemination: results of the randomised PERISCOPE II trial. Ann Oncol 2025; 36(Suppl 2): S1102–S1103

