# Supplementary material for: Management of gastric cancer peritoneal metastasis: International Gastric Cancer Association GCPM Working Group consensus statements
Source: Br J Surg. 2026 Mar 17;113(4):znag027. doi: 10.1093/bjs/znag027 (PMC13082581; doi:10.1093/bjs/znag027)
Supplement: znag027_Supplementary_Data [file znag027_supplementary_data.docx]

**Title:** Management of Gastric Cancer Peritoneal Metastasis: IGCA Working Group Consensus Statements

Authors

Piers R Boshier^1^, Daryl Kai Ann Chia^2^, Sri Ganeshamurthy Thrumurthy^2^, Jun Liang Teh^2^, Maria Wobith^2^, Maria Bencivenga^3^, Federica Filippini^3^, Teodora Dumitra^4^, Miguel Burch^4^, Hyoung-Il Kim^5^, Benjamin Kobitzsch^6^, Liudmila L Kodach^7^, Judith S E Quik^8^, Vo Duy Long^9^, Micha J de Neijs^10^, Pieter C van der Sluis^10^, Alberto M Leon-Takahashi^11^, Yanghee Woo^12^, Mickael Chevallay^13^, Massimo Framarini^14^, Paolo Morgagni^14^, Ewelina Frejlich^15^, Heike I Grabsch^16,17^, Sheraz R Markar^18^, Daniele Marrelli^19^, Do Joong Park^20^, Raghav Sundar^21^, Zekuan Xu^22^, Kay Khine Linn^23^, Han Kwang Yang^24^, Joji Kitayama^25^, Zhenggang Zhu^26^, Sun Young Rha^27^, Bas Wijnhoven^10^, Hiroharu Yamashita^28^, Wei Peng Yong^2^, Christelle de la Fouchardière^29^, Magnus Nilsson^30^, Hironori Ishigami^31^, Johanna W Van Sandick^7^, Florian Lordick^6^*, Brian D Badgwell^32^*, Jimmy Bok-Yan So^2,23,33^* on behalf of the IGCA GCPM Working Group.

^1^ Department of Surgery and Cancer, Imperial College London, UK

^2^ Division of General Surgery, National University Hospital, Singapore

^3^ Department of Surgery, Verona University, Verona, Italy

^4^ Department of Surgery, Cedars-Sinai Medical Center, Los Angeles, CA, USA

^5^ Department of Surgery, Yonsei University, Seoul, South Korea

^6^ Department of Medicine, University Cancer Center Leipzig, Leipzig University Medical Center,

Cancer Center Central Germany, Leipzig, Germany

^7^ Department of Surgical Oncology, The Netherlands Cancer Institute - Antoni van Leeuwenhoek

Hospital, Amsterdam, The Netherlands

^8^ Department of Pathology, The Netherlands Cancer Institute - Antoni van Leeuwenhoek

Hospital, Amsterdam, The Netherlands

^9^ Division of Upper GI Surgery, Department of GI surgical, University Medical Center, Ho Chi

Minh City, Vietnam

^10^ Department of Surgery, Erasmus University Medical Center, Rotterdam, Netherlands

^11^ Department of Surgical Oncology, National Cancer Institute, Mexico City, Mexico

^12^ Division of Surgical Oncology, Department of Surgery, City of Hope, Duarte and Orange

County, California, USA

^13^ Department of Surgery, Division of Visceral Surgery, University of Geneva, Switzerland

^14^ Department of Surgery, Morgagni-Pierantoni Hospital, Forlì, Italy

^15^ Department of Surgical Oncology, Faculty of Medicine, Wroclaw Medical University, Poland

^16^ Division of Pathology & Data Analytics, Leeds Institute of Medical Research at St. James’s,

University of Leeds, Leeds, UK

^17^ Department of Pathology, GROW Research Institute for Oncology and Reproduction,

Maastricht University Medical Center+, Maastricht, The Netherlands

^18^ Nuffield Department of Surgical Sciences, University of Oxford, UK

^19^ Department of Medicine, Surgery and Neurosciences, University of Siena, Italy

^20^ Department of Internal Medicine, Seoul National University Hospital, Seoul, South Korea

^21^ Department of Internal Medicine, Section of Medical Oncology, Yale School of Medicine, New

Haven, Connecticut, USA

^22^ Department of Surgery, Division of Gastric Surgery, the First Affiliated Hospital of Nanjing

Medical University, China

^23^ Department of Surgery, National University of Singapore, Singapore

^24^ Department of Surgery, Seoul National University Cancer Hospital, Seoul National University

Hospital, Seoul, South Korea

^25^ Department of Surgical Oncology, Japan Institute for Health Security, Japan

^26^ Department of Gastrointestinal Surgery, Rui Jin Hospital, Shanghai Jiao Tong University

School of Medicine, Shanghai, China

^27^ Department of Internal Medicine, Yonsei University College of Medicine, Songdang Institute for

Cancer Research, Yonsei University Health System, Seoul, Korea

^28^ Department of Surgery, Division of Gastroenterological, General and Transplant Surgery, Jichi

Medical University, Shimotsuke, Japan

^29^ Department of Medical Oncology, Institut Paoli-Calmettes, Marseille, France

^30^ Department of Upper Abdominal Diseases, Karolinska University Hospital and Department of

Clinical Science, Intervention and Technology, Karolinska Institutet, Stockholm, Sweden

^31^ Department of Chemotherapy, The University of Tokyo, Japan

^32^ Department of Surgical Oncology, Division of Surgery, The University of Texas MD Anderson

Cancer Center, Houston, Texas, USA

^33^ Division of Surgical Oncology, National University Cancer Institute, Singapore

**Corresponding author.** Jimmy Bok-Yan So

Department of Surgery, National University of Singapore, Singapore

**ORCID ID**; <https://orcid.org/0000-0002-9772-7905>

**Supplementary Materials - Index**

| **Supplementary Methods** |  |
| --- | --- |
| Consensus questions, evidence template | *page 3* |
|  |  |
| **Supplementary Results** |  |
| Summary of evidence and proposed consensus statements | *page 4* |
|  |  |
| **Supplementary Appendixes** |  |
| - | *page 14* |
|  |  |
| **Supplementary Figures and Tables** |  |
| **Table s1.** Statements submitted for first and second round voting including proposed revisions | *page 15* |
| **sFigure 1.** Results of voting for the first Delphi round amongst GCPM working group members | *page 18* |
|  |  |
| **References** | *page 19* |

**Supplementary Methods**

**IGCA GCPM working group**

**Consensus questions, evidence template**

Version 1.0

16^th^ September 2024

| **Consensus question:** |
| --- |
| **Method(s) use to obtain evidence**: |
| **Summary of evidence:** |
| **Consensus statement:** |
| **Level of evidence** *(Please refer to GRADE classification below)* |

**Classification of the Level of Evidence According to the GRADE System**

| **Type of study** | **A priori quality level** | **Decreases if** | **Increases if** | **A posteriori quality level** |
| --- | --- | --- | --- | --- |
| **Randomised studies** | **High** | ***Risk of bias*** | ***Effect*** | **High** |
|  |  | −1 significant | +1 large |  |
|  |  | −2 very significant | +2 very large |  |
|  |  | ***Inconsistency*** | ***Dose–response*** | **Moderate** |
|  |  | −1 significant | +1 obvious gradient |  |
|  |  | −2 very significant |  |  |
|  | | | | |
| **Observational studies** | **Low** | ***No direct evidence*** | ***All confounding factors:*** | **Low** |
|  |  | −1 significant | +1 would reduce observed effect |  |
|  |  | −2 very significant |  |  |
|  |  | ***Imprecision*** | +1 would suggest a spurious effect if there is no observed effect |  |
|  |  | −1 important |  | **Very low** |
|  |  | −2 very important |  |  |
|  |  | ***Publication bias*** |  |  |
|  |  | −1 likely |  |  |
|  |  | −2 very likely |  |  |

**Supplementary Results**

**IGCA GCPM working group**

**Summary of evidence and**

**proposed consensus statements**

***Statement 1.1, predictive factors*:** Key risk factors for GCPM include tumour histology, with diffuse-type and signet ring cell carcinoma showing a predisposition for peritoneal spread due to loss of cell adhesion (CDH1, CTNNA1 mutations) and epithelial-to-mesenchymal transition (EMT)(1-6). Molecular subtypes like TCGA Genomically Stable and ACRG EMT show elevated GCPM risk due to mutations and EMT-related pathways (5, 6). Emerging evidence suggests tumour-induced mesothelial activation and exosome secretion promote peritoneal niche formation, further aiding metastasis (7-9).

Tumour stage, specifically serosal invasion (T4a)(2-4) and advanced nodal metastasis (N2/N3)(1, 3), increase GPPM risk by facilitating direct or lymphatic spread. Positive peritoneal cytology indicates early microscopic dissemination, predicting future macroscopic GCPM (1). Younger age, female sex, and East Asian ethnicity correlate with higher diffuse-type cancers therefore GCPM incidence.(1, 3, 4, 10). Tumours of the body and distal stomach were also more likely to metastasise to the peritoneum (11).

*Discussion and amendments*: It request was made for ethnicity to be added to the statement.

| **Statement 1.1** |
| --- |
| Multiple patient specific (female sex; younger age, and; ethnicity) and tumour specific (T4a; cN2+; positive peritoneal cytology; diffuse-type; presence of SRC; genomically stable (TCGA); EMT subtype (ACRG); CLDN18.2-ARHGAP overexpression, and; mutations in CDH-1; CTNNA 1 and RHOA genes) risk factors of for peritoneal carcinomatosis have been acknowledged.  **Level of evidence**: Moderate / Very high; **Agreement** 95%; **CV** 0.16 |

***Statement 1.2, diagnosis*:** Staging laparoscopy is considered the gold standard for detecting GCPM offering high specificity (100%)(12-15) and the advantage of directly assessing GCPM extent. Whilst widely used Staging laparoscopy remains however, invasive and costly. Transcutaneous biopsy offers a less invasive alternative with excellent accuracy (93% sensitivity, 86% specificity) but cannot determine disease extent (16). Computer tomography often fails to detect small or diffuse GCPM deposits and is further limited by the expertise of the reporting radiologist accounting for its variable sensitivity (25–88%)(14, 17, 18). Peritoneal cytology is prognostically important but has highly variable diagnostic accuracy (19, 20).

*Discussion and amendments*: The original statement referenced MRI and FAPI-PET as methods to detect GCPM. It was however considered that these modalities were not commonly used or widely available for this purpose. As such it was considered that it would be more appropriate for these imaging modalities to be added to Statement 3.1 that describes novel diagnostics.

| **Statement 1.2** |
| --- |
| Computer tomography is a suitable non-invasive and accessible first line investigation for GCPM. Where possible and appropriate (e.g. in patients with potentially resectable disease), staging laparoscopy with peritoneal cytology and histopathology of peritoneal deposits remains the gold standard for diagnosing GCPM.  **Level of evidence**: Low / Very low; **Agreement** 90%; **CV** 0.15 |

***Statement 1.3, classification*:** Reliable grading of GCPM is essential for estimating the probability of achieving complete cytoreductive surgery, forecasting prognosis, and selecting candidates for intensified multimodal therapy (21). The Japanese P1/2/3 classification system, first proposed in 1981 (22), was refined as P1a/b/c system in 2017 (23). This semi-quantitative approach categorizes disease spread across three broad levels. In contrast, the Peritoneal carcinomatosis index, introduced by Sugarbaker in 1996, is a fully quantitative scoring system (ranging from 0-39) and remains the most widely adopted tool for peritoneal carcinomatosis across various cancer types (24). Such classification systems are considered crucial for assessing disease extent and predicting the feasibility of complete cytoreduction.

*Discussion and amendments*: Amendments were made attributing peritoneal carcinoma index (PCI) to Sugarbaker and updating the latest definitions of the Japanese classification system. Further requests at the time of the IGCC consensus session were made to remove an accompanying statement referring to the evaluation of the completeness of surgical resection.

| **Statement 1.3** |
| --- |
| For patients who are being considered for multimodal treatment of GCPM, quantitative classification of peritoneal metastasis, using either PCI (Sugarbaker) or P1a/b/c (Japanese) classification systems, is crucial for assessing disease extent and predicting the feasibility of complete cytoreduction.  **Level of evidence**: Low; **Agreement** 100%; **CV** 0.11 |

***Statement 1.4, definition of limited GCPM*:** Studies support using a PCI ≤6 as the threshold for limited peritoneal disease, correlating it with improved prognosis and overall survival (OS)(25-37). Most trials demonstrated consistent findings through both univariate and multivariate analyses, showing lower PCI as an independent predictor of better OS and disease-free survival (DFS). Other studies had less conclusive results, with PCI not always statistically significant in univariate and multivariate analysis (38, 39). Differentiation between poorly cohesive carcinoma (PCC) and non-PCC patients has suggested stratified PCI cut-offs for each subtype (40).

The threshold for limited GCPM has been examined in a single meta-analysis published in 2015 (41). Whilst a PCI <12 was associated with better 1-5 year survival, this finding may be considered outdated in light on more recent evidence outline above (41).

*Discussion and amendments*: inclusion of P1a, from the Japanese classification system, in the definition of limited GCPM

| **Statement 1.4** |
| --- |
| The definition of limited peritoneal metastases could be PCI ≤ 6 (Sugarbaker classification) or P1a (Japanese classification), but specification of the definition for subgroups may help to work towards more patient tailored treatment.  **Level of evidence**: Low; **Agreement** 90%; **CV** 0.13 |

***Statement 2.1, systemic chemotherapy*:** Evidence on response rates to systemic treatment (ST) only can be gained from three phase III trial (42-44), six phase II trials (45-50) and two retrospective analysis (51, 52). Four trials used a three-drug regimen (42, 45, 47, 49) while the others applied two-drug combinations (43, 44, 46, 48, 50, 52). Complete peritoneal response rates were between 19% and 40% while response assessed by RECIST was between 25% and 60%. RECIST response data is only available for a small subset of patients due to limitations on evaluating GCPM by computed tomography.

Evidence on the optimal systemic treatment for GCPM remains limited. Recent large phase III trials (e.g., Checkmate 649, GLOW, Keynote 811/859, SPOTLIGHT)(53-57) did not report outcomes specifically for GCPM patients. First-line trials exclusively involving GCPM patients focused on those with very advanced disease (58-60) or failed to meet primary endpoints (61). Real-world data from the Netherlands suggest improved survival with triplet chemotherapy including docetaxel (e.g., FLOT) over standard doublet regimens (FOLFOX or CAPOX)(51). Second-line treatment studies included some regimens that do not always represent the globally recommended standard of care in 2025 (62-66).

Median overall survival with systemic therapy alone ranged from 13.0 to 15.2 months in three key trials using either FLOT (45), cisplatin/S-1 (43), EOX or cisplatin / fluorouracil / trastuzumab (42).

No studies have explored the optimal duration of systemic therapy.

Data on targeted and immunotherapy is scarce, with studies on tislelizumab (67) and ramucirumab (61) showing no significant difference between gastric cancer patients with or without GCPM. However, real-world evidence supports adding trastuzumab to doublet chemotherapy for improved outcomes (51). Whilst treatment of CLND18.2-positive metastatic gastric cancer with Zolbetuximab was beneficial, results were not specific for GCPM (54, 57).

*Discussion and amendments*: this remained one of the more debated consensus statements largely due to the variation in systemic chemotherapy regime used across different centres and regions. It was deemed important that the statement be amended to clarify that that the reference point for the efficacy of systematic chemotherapy should be best supportive care.

| **Statement 2.1** |
| --- |
| Optimal drug regimens and duration of treatment in GCPM have not been defined. In addition to best supportive care alone, systemic chemotherapy, often a combination of platinum compounds, fluoropyrimidines and taxanes, remains the standard of care for patient with GCPM. Use of targeted- and immune-therapies should be to individualised to patients.  **Level of evidence**: High* / Very low**; **Agreement** 81%; **CV** 0.26  *Concerning evidence for the overall survival; **Concerning evidence for optimal duration to systemic chemotherapy |

***Statement 2.2, intraperitoneal chemotherapy*:** Intraperitoneal chemotherapy (IPC) for GCPM includes three main methods: hyperthermic intraperitoneal chemotherapy (HIPEC); pressurised intraperitoneal aerosolised chemotherapy (PIPAC), and; normothermic intraperitoneal chemotherapy. HIPEC shows some survival benefits in retrospective studies, but prospective trials like GASTRIPEC-I and PERISCOPE II have been inconclusive or negative for overall survival (42, 68). The PERISCOPE II Trial found that gastrectomy and cytoreductive surgery with HIPEC resulted in higher rates of serious adverse events and provided no survival benefit in patients with gastric cancer and limited peritoneal metastasis compared to systemic therapy alone (68). PIPAC has shown promise in observational studies, but its only randomized trial (EstoK-01) was stopped early due to toxicity without clear efficacy (69). Normothermic intraperitoneal chemotherapy with systemic chemotherapy (NIPS), especially using taxanes like paclitaxel, has the strongest evidence. Trials such as PHOENIX-GC (43) and DRAGON-01(44) suggest improved survival and control of ascites, with DRAGON-01 demonstrating a significant overall survival benefit. Recommended IP agents vary by method, including oxaliplatin and cisplatin for HIPEC, cisplatin and doxorubicin for PIPAC, and paclitaxel or docetaxel for CIPC. Overall, Normothermic IPC with taxanes currently offers the most promising and evidence-supported intraperitoneal chemotherapy chemotherapy option for GCPM.

*Discussion and amendments*: after discussion minor typographic changes were made to the statement to improve meaning. No other amendments were requested.

| **Statement 2.2** |
| --- |
| Intraperitoneal chemotherapy administered by the means of HIPEC or PIPAC are still investigational. While normothermic intraperitoneal paclitaxel can be recommended in clinical practice in Eastern Asian populations, Western trials are still needed for the sake of generalizability.  **Level of evidence**: HIPEC: Low; PIPAC: Low; normothermic IP paclitaxel: Moderate / High **Agreement** 86%; **CV** 0.13 |

***Statement 2.3, factors predicting efficacy of intraperitoneal chemotherapy*:** Several clinical factors may influence the efficacy of IPC in GCPM. Patient performance status, ECOG score of 1 or improvement in the Karnofsky Performance Scale after treatment, predicts better survival (70, 71).

The RECIST criteria are limited in assessing peritoneal metastases because these lesions are frequently difficult to assess and measure accurately. As a result, alternative evaluation methods that focus on the overall extent and distribution of peritoneal tumour burden provide a more reliable assessment of disease status in such cases. The Japanese Classification of Gastric Carcinoma has however shown inconsistent predictive value due to low numbers of patients with limited (P1a) disease in many studies (43, 72). P1a/b/c staging has been shown to be superior to P1/2/3 and Gilly staging in predicting the survival of GCPM patients (73). Several studies suggest better survival and suitability for conversion surgery when PCI is ≤12, with optimal outcomes at PCI ≤6-7 (74). Higher volumes of malignant ascites were associated with worse outcomes (43, 70, 71). Although PCI wasn’t an independent factor in some analyses, cytoreductive surgery plus HIPEC showed survival benefits even in high PCI (>20) cases (75). Finally, achieving complete cytoreduction (CC 0-1) after surgery correlates with improved prognosis, highlighting its importance in treatment success (75, 76).

*Discussion and amendments*: the statement was amended to include reference to the finding that the presence of malignant ascites is a prognostic marker in patients receiving IPC.

| **Statement 2.3** |
| --- |
| Multiple factors including patient performance status, presence of malignant ascites, peritoneal disease burden, tumour chemosensitivity, treatment regimen and mode of delivery may predict efficacy of intraperitoneal chemotherapy.  **Level of evidence**: Low; **Agreement** 95%; **CV** 0.12 |

***Statement 2.4, therapeutic response*:** Radiological assessment, CT and RECIST criteria, is commonly used but its relationship with overall survival is not well studied.

PCI has been shown to impact overall survival (25-37) but can only be assessed in the setting of laparoscopy, or if cytoreductive/conversion surgery is being performed. Laparoscopic PCI may however under stage GCPM burden (77). At the time of laparoscopy cytology and peritoneal biopsies can be taken and evaluated. Conversion to negative cytology or higher regression of GCPM may have a favourable prognostic impact (78). Assessment of cytology likely has a high specificity but variable (low sensitivity)(20). A Peritoneal Regression Grading Score (PRGS) of 1, where no tumour cells are found within peritoneal biopsies is associated with better survival, but may need confirmation with additional immunohistochemical stains (79). Pathological response of the primary tumour (in synchronous GCPM) may also be of prognostic value.

A number of patient reported outcome tools are available but burden of administering questionnaires to patients should be considered.

*Discussion and amendments*: The statement was amended to place survival ahead of radiological response as the key metric of therapeutic response in patients with GCPM. Reference to the importance of PRGS was also added.

| **Statement 2.4** |
| --- |
| Survival and radiological response remain the most widely used metrics of therapeutic response in patients with GCPM. In cases where conversion surgery is being considered further evaluation by laparoscopy and the Peritoneal Regression Grading Score (PRGS) should be undertaken allowing for more accurate evaluation of GCPM burden. Greater emphasis on patient reported outcomes is also necessary.  **Level of evidence**: Low / Very low; **Agreement** 95%; **CV** 0.12 |

***Statement 2.5, conversion surgery*:** Eleven retrospective studies showed that conversion surgery significantly improved median survival time (16 to 43 months) compared to chemotherapy alone (8 to 12 months). Patients achieving complete (R0) resection had the best outcomes, with a 3-year survival of 65% (80-90). A further 11 studies evaluated conversion surgery combined with IPC (including HIPEC)(91-101). The addition of IPC generally improved survival, with median overall survival ranging from 13 to 47 months. Separately, 18 studies focused on HIPEC, showing that surgery plus HIPEC often led to better survival than surgery or HIPEC alone (25, 27, 28, 30, 32, 40, 42, 102-112). However, survival outcomes varied across studies. Recently The PERISCOPE II Trial found that gastrectomy and cytoreductive surgery with HIPEC resulted in higher rates of serious adverse events and provided no survival benefit in patients with gastric cancer and limited peritoneal metastasis compared to systemic therapy alone (68). The IKF-575/RENAISSANCE Study notably showed that in patients with peritoneal metastasis who received chemotherapy/targeted therapy the addition of surgery was detrimental to overall survival (113).

Definitions of peritoneal disease burden differed, but lower burden (P1 or PCI <10–12) was associated with better outcomes after conversion surgery.

Overall, conversion surgery after chemotherapy improves survival, but the added benefit of intraperitoneal therapies remains uncertain due to limited, heterogeneous evidence and small sample sizes.

*Discussion and amendments*: The statement was amended to emphasise that conversion surgery “maybe” therapeutic option for selected patients with limited GCPM. Reference to bidirectional therapy was removed.

| **Statement 2.5** |
| --- |
| Conversion surgery maybe a therapeutic option for selected patients with limited GCPM. The impact of adding HIPEC at time of conversion surgery warrants further investigation.  **Level of evidence**: Low; **Agreement** 81%; **CV** 0.20 |

***Statement 2.6, palliative care*:** Oncology societies emphasise the critical role of palliative care in managing patients with cancer, including those with GCPM. The ASCO guidelines, updated in May 2024, recommend early referral to specialised interdisciplinary palliative care teams alongside active treatment for patients with advanced solid tumours, especially those with uncontrolled symptoms or quality-of-life issues (114). These guidelines address effective interventions, appropriate patient referrals, and quality-of-life outcomes but notably lack surgical input. The National Comprehensive Cancer Network highlights palliative care goals as optimal symptom management, psychosocial-spiritual support, and reducing suffering while enhancing quality of life for patients and caregivers (115). Publications on GCPM and palliative care, mainly address gastric outlet obstruction, with few focusing specifically on carcinomatosis or malignant bowel obstruction. Outcomes varied widely, with no randomised trials or surgical studies addressing palliative care consensus. Key unmet needs include managing malignant bowel obstruction, malignant ascites, and fostering interdisciplinary care, indicating areas requiring further research.

*Discussion and amendments*: no revisions to the statement were request.

| **Statement 2.6** |
| --- |
| Palliative care should provide the best symptom management possible for the patient and their family/caregivers, with the objective of providing optimal quality of life in the context of their goals of care.  **Level of evidence**: Low; **Agreement** 100%; **CV** 0.07 |

***Statement 2.7, prophylactic treatment*:** Ten randomised (116-125) and five observational studies (126-130) on prophylactic HIPEC show potential benefits, such as improved 3-year survival and reduced peritoneal recurrence, especially in high-risk patients. Prophylactic use of HIPEC was not generally associated with increased the morbidity or mortality. However, limitations of these studies included their small sample size and heterogenous methods with most of the studies originating from Asia.

Evidence for prophylactic normothermic IPC is inconclusive, with mixed survival outcomes and some increased complications (131-134). Prophylactic PIPAC’s is a relatively novel approach, with one recent small study showing that its use is feasible and safe after laparoscopic gastrectomy (135). Further well-designed randomised trials, including ongoing phase III studies (GASTRICHIP, GOETH, PREVENT, DRAGON II, PHOENIX-GC2), are needed to clarify efficacy, safety, and patient selection.

*Discussion and amendments*: Feedback led to amendment of the statement to emphasise the use of “intraperitoneal treatments” in GCPM prophylaxis. These treatments were widely considered to be “experimental” for GCPM prophylaxis indicating that “further clinical trials” are needed.

| **Statement 2.7** |
| --- |
| While prophylactic intraperitoneal treatments show promise in reducing peritoneal metastasis and improving survival outcomes in high-risk gastric cancer populations, this application is still experimental, and further clinical trials are needed to establish the efficacy across diverse populations and the impact on survival.  **Level of evidence**: Low / Very low; **Agreement** 100%; **CV** 0.10 |

***Statement 3.1*, *novel diagnostics*:** Emerging diagnostic methods fall into three categories: Advanced computational models, primarily analysing CT images may enable non-invasive detection, staging, and monitoring of GCPM by identifying radiographic features correlating with disease burden (136-143). Other non-machine learning radiomic models show promising sensitivity (>80%) and specificity (>75%)(18). PET imaging, especially with fibroblast activation protein inhibitor (FAPI), has improved detection sensitivity for peritoneal metastases compared to traditional methods (144, 145). Magnetic resonance imaging (MRI) is another non-invasive diagnostic tool, and its sensitivity and specificity appear to offer slightly higher diagnostic accuracy compared to CT scans (146, 147). Preclinical models using bioluminescence and fluorescence allow real-time monitoring of GCPM progression (148, 149). Finally, genetic and protein markers, including circulating tumour DNA, miRNA, and proteomic profiles from peritoneal fluid, provide molecular signatures for detecting GCPM and assessing prognosis (150-155). Cancer marker kinetics (e.g., CA125, CA72-4) also correlate with disease progression and treatment response (156, 157). Together, these innovative tools have the potential to improve future early detection, monitoring, and personalised treatment of GCPM.

*Discussion and amendments*: please see comment for statement 1.2.

| **Statement 3.1** |
| --- |
| Fibroblast activation protein inhibitor positron emission tomography (FAPI-PET) and diffusion-weighted magnetic resonance (DW-MRI) are emerging imaging modalities for the assessment of GCPM. Additionally, confirmatory testing of peritoneal fluid for genetic and protein biomarkers, offers a feasible and minimally invasive strategy to evaluate progression or regression of GCPM.  **Level of evidence**: Low / Very low; **Agreement** 95%; **CV** 0.11 |

***Statement 3.7, novel therapeutics*:** Innovative therapeutic approaches for GCPM focus on the tumour’s unique microenvironment and include five main categories:

1. *Targeted Therapies:* Various micro-RNAs have shown promise in suppressing GCPM in mouse models (158-162). Intraperitoneal immunotherapies like catumaxomab and trastuzumab have been tested in humans, while anti-PD-1 treatments have shown efficacy in animal studies (163-165).
2. *Oncolytic Viruses:* Virotherapy aims to kill tumour cells and stimulate immune responses. Though no human trials exist for GCPM yet, mouse studies show potential (166). A Phase I/II trial using a novel HSV-2-derived oncolytic virus demonstrated durable antitumor activity in other cancers (167).
3. *Direct Therapies:* Approaches such as intraperitoneal α-particle therapy (168), photodynamic (169), and photothermal therapies target peritoneal metastases directly. An early human trial of α-emitting microparticles in postoperative colorectal patients demonstrated an encouraging safety profile (170-172).
4. *Enhancement of Intraperitoneal Therapy:* Techniques include hydrogels for sustained drug release, electrostatic forces to improve chemotherapy distribution (e.g., ePIPAC)(173, 174), and nanoparticles like nab-paclitaxel for drug delivery (175). These show promise but require more human studies.
5. *Alternative Therapies:* Traditional Chinese herbal medicines, such as Yangzheng Xiaoji and Elemene, have some preclinical evidence suggesting benefit in treating peritoneal metastases (176-178).

Overall, these novel strategies remain experimental supported by low evidence but offer potential avenues for future GCPM treatments.

*Discussion and amendments*: no revisions to the statement were request.

| **Statement 3.2** |
| --- |
| Novel therapeutic strategies for GCPM should seek to leverage the unique anatomy and tumour microenvironment of the peritoneum including development of enhanced regional and targeted therapies.  **Level of evidence**: Very low; **Agreement** 95%; **CV** 0.13 |

**Supplementary Appendixes**

**Supplementary Figures and Tables**

**Table s1.** Statements submitted for first and second round voting including proposed revisions

| **ID** | **Question** | **Consensus statements revisions after 1^st^ Round voting** (final statements with revisions are found within the manuscript) | **Evidence** | **Voting, 1^st^ Round**  30^th^ April to 6^th^ May 2025  No. voting n=28 | **Voting, 2^st^ Round**  9^th^ May 2025 |
| --- | --- | --- | --- | --- | --- |
| 1.1 | What factors predict risk of GCPM? | [**Proposed**] Patient specific risk factors for peritoneal carcinomatosis from gastric cancer include: female sex; younger age, and; East Asian ethnicity. Tumour specific risk factors for peritoneal carcinomatosis: T4a; cN2+; positive peritoneal cytology; diffuse-type; presence of SRC; genomically stable (TCGA); EMT subtype (ACRG); CDH-1 mutation carrier; CTNNA 1 carrier; RHOA mutation carrier, and; CLDN18.2-ARHGAP overexpression.  [**Revised_1**] Patient specific risk factors for peritoneal carcinomatosis from gastric cancer include: female sex; younger age; Tumour specific risk factors for peritoneal carcinomatosis: T4a; cN2+; positive peritoneal cytology; diffuse-type; presence of SRC; genomically stable (TCGA); EMT subtype (ACRG); CDH-1 mutation carrier; CTNNA 1 carrier; RHOA mutation carrier, and; CLDN18.2-ARHGAP overexpression. | Moderate to High | Strongly agree=29%  Agree=54%  Neutral=11%  Disagree=7%  Strongly disagree=0%  Agreement 83%  Consensus reached: **Yes** | No. Voting n= **62**  Strongly agree=23%  Agree=76%  Neutral=0%  Disagree=2%  Strongly disagree=0%  Agreement 99%  Consensus reached: **Yes** |
| 1.2 | By which method(s) should GCPM be diagnosed? | [**Proposed**] In addition to CT, staging laparoscopy with peritoneal cytology and where possible histopathology of peritoneal deposits remains the gold standard for diagnosing GCPM. FAPI-PET and MRI have the potential to be accurate minimally invasive alternatives.  [**Revised_1**] In addition to CT, staging laparoscopy with peritoneal cytology and where possible histopathology of peritoneal deposits remains the gold standard for diagnosing GCPM. MRI and others such as FAPI-PET may be potential imaging modalities. | Low to very low | Strongly agree=46%  Agree=50%  Neutral=4%  Disagree=0%  Strongly disagree=0%  Agreement 96%  Consensus reached: **Yes** | No. Voting n= **63**  Strongly agree=68%  Agree=25%  Neutral=6%  Disagree=0%  Strongly disagree=0%  Agreement 93%  Consensus reached: **Yes** |
| 1.3 | By what method is GCPM best classified? | [**Proposed**] For patients who are being considered for multimodal treatment of GCPM, quantitative classification of peritoneal metastasis, using either PCI or Japanese (P1a/b/c,2,3) classification systems, is crucial for assessing disease extent and predicting the feasibility of complete cytoreduction.  Completeness of surgical resection can be evaluated using CC scores and R scores, reflecting residual disease.  [**Revised_1**] *For patients who are being considered for multimodal treatment of GCPM, quantitative classification of peritoneal metastasis, using either PCI (Sugarbaker) or Japanese (P1a/b/c) classification systems, is crucial for assessing disease extent and predicting the feasibility of complete cytoreduction.*  *Completeness of surgical resection can be evaluated using CC scores, reflecting residual disease* | Low | Strongly agree=29%  Agree=68%  Neutral=4%  Disagree=0%  Strongly disagree=0%  Agreement 97%  Consensus reached: **Yes** | No. Voting n= **65**  Strongly agree=49%  Agree=46%  Neutral=5%  Disagree=0%  Strongly disagree=0%  Agreement 95%  Consensus reached: **Yes** |
| 1.4 | What is the definition of limited GCPM? | [**Proposed**] The definition of limited peritoneal metastases could be PCI ≤ 6, but specification of the definition PCI for subgroups may help to work towards more patient tailored treatment.  [**Revised_1**] No change | Low | Strongly agree=18%  Agree=71%  Neutral=4%  Disagree=7%  Strongly disagree=0%  Agreement 89%  Consensus reached: **Yes** | No. Voting n= **64**  Strongly agree=19%  Agree=53%  Neutral=25%  Disagree=3%  Strongly disagree=0%  Agreement 72%  Consensus reached: **Yes** |
| 2.1 | Is systemic chemotherapy when given alone (without intraperitoneal chemotherapy) an effective treatment for GCPM? | [**Proposed**] Optimal drug regimens and duration of treatment in GCPM have not been defined. Systemic chemotherapy, often a combination of platinum compounds, fluoropyrimidines and taxanes, however remains the standard of care for patient with GCPM. Use of targeted- and immune-therapies should be to individualised to patients.  [**Revised_1**] No change | High to low | Strongly agree=32%  Agree=43%  Neutral=18%  Disagree=7%  Strongly disagree=0%  Agreement 75%  Consensus reached: **Yes** | No. Voting n= **65**  Strongly agree=9%  Agree=42%  Neutral=17%  Disagree=26%  Strongly disagree=6%  Agreement 51%  Consensus reached: **Yes** |
| 2.2 | Is intraperitoneal chemotherapy an effective treatment for GCPM? | [**Proposed**] Intraperitoneal chemotherapy administered by the means of HIPEC or PIPAC are still investigational, while catheter administered paclitaxel may be recommended in clinical practice in Eastern Asian populations, while Western trials are still needed for the sake of generalizability*.*  [**Revised_1**] Intraperitoneal chemotherapy administered by the means of HIPEC or PIPAC are still investigational, while catheter administered paclitaxel may be recommended in clinical practice in Eastern Asian populations, Western trials are still needed for the sake of generalizability*.* | HIPEC: low  PIPAC: low  Normo-thermic IP paclitaxel: moderate to high | Strongly agree=18%  Agree=82%  Neutral=0%  Disagree=0%  Strongly disagree=0%  Agreement 100%  Consensus reached: **Yes** | No. Voting n= **66**  Strongly agree=11%  Agree=71%  Neutral=18%  Disagree=0%  Strongly disagree=0%  Agreement 82%  Consensus reached: **Yes** |
| 2.3 | What factors predict efficacy of intraperitoneal chemotherapy? | [**Proposed**] Multiple factors including patient perfomance status, peritoneal disease burden, tumour chemosensitivity, treatment regimen and mode of delivery may predict efficacy of intraperitoneal chemotherapy.  [**Revised_1**] Multiple factors including patient perfomance status, presence of malignant ascites, peritoneal disease burden, tumour chemosensitivity, treatment regimen and mode of delivery may predict efficacy of intraperitoneal chemotherapy. | Low | Strongly agree=25%  Agree=64%  Neutral=11%  Disagree=0%  Strongly disagree=0%  Agreement 89%  Consensus reached: **Yes** | No. Voting n= **63**  Strongly agree=19%  Agree=78%  Neutral=3%  Disagree=0%  Strongly disagree=0%  Agreement 97%  Consensus reached: **Yes** |
| 2.4 | How should GCPM therapeutic response be evaluated? | [**Proposed**] Radiological response and survival remain the most widely used metrics of therapeutic response in patients with GCPM. In cases where conversion surgery is being considered further evaluation by laparoscopy should be undertaken allowing for more accurate evaluation of GCPM burden. There is also a need for greater emphasis on patient reported outcomes.  [**Revised_1**] Survival and radiological response remain the most widely used metrics of therapeutic response in patients with GCPM. In cases where conversion surgery is being considered further evaluation by laparoscopy should be undertaken allowing for more accurate evaluation of GCPM burden. There is also a need for greater emphasis on patient reported outcomes. | Low to very low | Strongly agree=22%  Agree=63%  Neutral=7%  Disagree=7%  Strongly disagree=0%  Agreement 85%  Consensus reached: **Yes** | No. Voting n= **63**  Strongly agree=21%  Agree=78%  Neutral=2%  Disagree=0%  Strongly disagree=0%  Agreement 99%  Consensus reached: **Yes** |
| 2.5 | Is there a role for conversion surgery in the treatment of GCPM? | [**Proposed**] Conversion surgery after systemic or bidirectional chemotherapy is a therapeutic option for selected patients with limited GCPM. The impact of adding HIPEC at time of conversion surgery warrants further investigation.  [**Revised_1**] Conversion surgery after systemic or bidirectional chemotherapy maybe a therapeutic option for selected patients with limited GCPM. The impact of adding HIPEC at time of conversion surgery warrants further investigation. | Low | Strongly agree=39%  Agree=43%  Neutral=14%  Disagree=0%  Strongly disagree=4%  Agreement 82%  Consensus reached: **Yes** | No. Voting n= **63**  Strongly agree=46%  Agree=44%  Neutral=8%  Disagree=2%  Strongly disagree=0%  Agreement 90%  Consensus reached: **Yes** |
| 2.6 | What should the objectives of palliative care in GCPM be? | [**Proposed**] Palliative care should provide the best symptom management possible for the patient and their family/caregivers, with the objective of providing optimal quality of life in the context of their goals of care.  [**Revised_1**] No change | Low | Strongly agree=75%  Agree=21%  Neutral=4%  Disagree=0%  Strongly disagree=0%  Agreement 96%  Consensus reached: **Yes** | No. Voting n= **61**  Strongly agree=89%  Agree=11%  Neutral=0%  Disagree=0%  Strongly disagree=0%  Agreement 100%  Consensus reached: **Yes** |
| 2.7 | What evidence is there for prophylactic treatment of GCPM ? | [**Proposed**] While prophylactic peritoneal treatments show promise in reducing peritoneal metastasis and improving survival outcomes in high-risk gastric cancer populations, this application is still under investigation, and further studies are needed to establish the efficacy across diverse populations and the impact on survival.  [**Revised_1**] While prophylactic peritoneal treatments show promise in reducing peritoneal metastasis and improving survival outcomes in high-risk gastric cancer populations, this application is still experimental, and further clinical trials are needed to establish the efficacy across diverse populations and the impact on survival. | Low to very low | Strongly agree=25%  Agree=61%  Neutral=11%  Disagree=4%  Strongly disagree=0%  Agreement 86%  Consensus reached: **Yes** | No. Voting n= **59**  Strongly agree=25%  Agree=71%  Neutral=3%  Disagree=0%  Strongly disagree=0%  Agreement 96%  Consensus reached: **Yes** |
| 3.1 | What are novel approaches for detecting, staging, and/or monitoring gastric cancer peritoneal metastases (GCPM)? | [**Proposed**] High-powered modelling and emission- or light-based imaging techniques provide novel avenues for improved visualization. Additionally, confirmatory testing of peritoneal fluid for genetic and protein biomarkers, is emerging as a feasible and minimally invasive strategy to evaluate progression or regression of GCPM.  [**Revised_1**] High-powered modelling and emission- or light-based imaging techniques may provide novel avenues for improved visualization. Additionally, confirmatory testing of peritoneal fluid for genetic and protein biomarkers, is emerging as a feasible and minimally invasive strategy to evaluate progression or regression of GCPM. | Very low | Strongly agree=14%  Agree=68%  Neutral=18%  Disagree=0%  Strongly disagree=0%  Agreement 82%  Consensus reached: **Yes** | No. Voting n= **58**  Strongly agree=16%  Agree=74%  Neutral=10%  Disagree=0%  Strongly disagree=0%  Agreement 90%  Consensus reached: **Yes** |
| 3.2 | What potential novel therapeutic strategies are there for treating GCPM? | [**Proposed**] Novel therapeutic strategies for GCPM should seek to leverage the unique anatomy and tumour microenvironment of the peritoneum including development of enhanced regional and targeted therapies.  [**Revised_1**] No change | Very low | Strongly agree=29%  Agree=54%  Neutral=18%  Disagree=0%  Strongly disagree=0%  Agreement 83%  Consensus reached: **Yes** | No. Voting n= **58**  Strongly agree=33%  Agree=60%  Neutral=5%  Disagree=2%  Strongly disagree=0%  Agreement 93%  Consensus reached: **Yes** |

**sFigure 1.** Results of voting for the first Delphi round amongst GCPM working group members

**References**

1. Huang B, Rouvelas I, Nilsson M. Gastric and gastroesophageal junction cancer: Risk factors and prophylactic treatments for prevention of peritoneal recurrence after curative intent surgery. Ann Gastroenterol Surg. 2022;6(4):474-85.

2. Seyfried F, von Rahden BH, Miras AD, Gasser M, Maeder U, Kunzmann V, et al. Incidence, time course and independent risk factors for metachronous peritoneal carcinomatosis of gastric origin--a longitudinal experience from a prospectively collected database of 1108 patients. BMC Cancer. 2015;15:73.

3. Agnes A, Biondi A, Persiani R, Laurino A, Reddavid R, De Giuli M, et al. Development of the PERI-Gastric (PEritoneal Recurrence Index) and PERI-Gram (Peritoneal Recurrence Index NomoGRAM) for predicting the risk of metachronous peritoneal carcinomatosis after gastrectomy with curative intent for gastric cancer. Gastric Cancer. 2022;25(3):629-39.

4. Glatz T, Verst R, Kuvendjiska J, Bronsert P, Becker H, Hoeppner J, et al. Pattern of Recurrence and Patient Survival after Perioperative Chemotherapy with 5-FU, Leucovorin, Oxaliplatin and Docetaxel (FLOT) for Locally Advanced Esophagogastric Adenocarcinoma in Patients Treated Outside Clinical Trials. J Clin Med. 2020;9(8).

5. Cristescu R, Lee J, Nebozhyn M, Kim KM, Ting JC, Wong SS, et al. Molecular analysis of gastric cancer identifies subtypes associated with distinct clinical outcomes. Nat Med. 2015;21(5):449-56.

6. Chen Y, Zhou Q, Wang H, Zhuo W, Ding Y, Lu J, et al. Predicting Peritoneal Dissemination of Gastric Cancer in the Era of Precision Medicine: Molecular Characterization and Biomarkers. Cancers (Basel). 2020;12(8).

7. Zhu M, Zhang N, Ma J, He S. Integration of exosomal miR-106a and mesothelial cells facilitates gastric cancer peritoneal dissemination. Cell Signal. 2022;91:110230.

8. Zhu AK, Shan YQ, Zhang J, Liu XC, Ying RC, Kong WC. Exosomal NNMT from peritoneum lavage fluid promotes peritoneal metastasis in gastric cancer. Kaohsiung J Med Sci. 2021;37(4):305-13.

9. Li Q, Li B, Li Q, Wei S, He Z, Huang X, et al. Exosomal miR-21-5p derived from gastric cancer promotes peritoneal metastasis via mesothelial-to-mesenchymal transition. Cell Death Dis. 2018;9(9):854.

10. Khan A, Ituarte PHG, Raoof M, Melstrom L, Li H, Yuan YC, et al. Disparate and Alarming Impact of Gastrointestinal Cancers in Young Adult Patients. Ann Surg Oncol. 2021;28(2):785-96.

11. Wang L, Liang B, Jiang Y, Huang G, Tang A, Liu Z, et al. Subsite-specific metastatic organotropism and risk in gastric cancer: A population-based cohort study of the US SEER database and a Chinese single-institutional registry. Cancer Med. 2023;12(19):19595-606.

12. Asencio F, Aguilo J, Salvador JL, Villar A, De la Morena E, Ahamad M, et al. Video-laparoscopic staging of gastric cancer. A prospective multicenter comparison with noninvasive techniques. Surg Endosc. 1997;11(12):1153-8.

13. Fujimura T, Kinami S, Ninomiya I, Kitagawa H, Fushida S, Nishimura G, et al. Diagnostic laparoscopy, serum CA125, and peritoneal metastasis in gastric cancer. Endoscopy. 2002;34(7):569-74.

14. Leeman MF, Patel D, Anderson J, O'Neill JR, Paterson-Brown S. Multidetector Computed Tomography Versus Staging Laparoscopy for the Detection of Peritoneal Metastases in Esophagogastric Junctional and Gastric Cancer. Surg Laparosc Endosc Percutan Tech. 2017;27(5):369-74.

15. Stell DA, Carter CR, Stewart I, Anderson JR. Prospective comparison of laparoscopy, ultrasonography and computed tomography in the staging of gastric cancer. Br J Surg. 1996;83(9):1260-2.

16. Souza FF, Mortele KJ, Cibas ES, Erturk SM, Silverman SG. Predictive value of percutaneous imaging-guided biopsy of peritoneal and omental masses: results in 111 patients. AJR Am J Roentgenol. 2009;192(1):131-6.

17. Burbidge S, Mahady K, Naik K. The role of CT and staging laparoscopy in the staging of gastric cancer. Clin Radiol. 2013;68(3):251-5.

18. Li ZY, Tang L, Li ZM, Li YL, Fu J, Zhang Y, et al. Four-Point Computed Tomography Scores for Evaluation of Occult Peritoneal Metastasis in Patients with Gastric Cancer: A Region-to-Region Comparison with Staging Laparoscopy. Ann Surg Oncol. 2020;27(4):1103-9.

19. Van Der Sluis K, Taylor SN, Kodach LL, van Dieren JM, de Hingh I, Wijnhoven BPL, et al. Tumor-positive peritoneal cytology in patients with gastric cancer is associated with poor outcome: A nationwide study. Eur J Cancer. 2024;199:113541.

20. Lisiecki R, Kruszwicka M, Spychała A, Murawa D. Prognostic significance, diagnosis and treatment in patients with gastric cancer and positive peritoneal washings. A review of the literature. Rep Pract Oncol Radiother. 2017;22(6):434-40.

21. Kim HI, Badgwell BD. Peritoneal Oligometastasis in Gastric Cancer: Diagnostic Strategies, Patient Selection, and Emerging Therapeutic Approaches. J Gastric Cancer. 2025;25(3):409-23.

22. Kajitani T. The general rules for the gastric cancer study in surgery and pathology. Part I. Clinical classification. Jpn J Surg. 1981;11(2):127-39.

23. Japanese Gastric Cancer Association. Japanese Classification of Gastric Carcinoma. 15th ed. Tokyo: Kanehara, 2017. 2017.

24. Jacquet P, Sugarbaker PH. Clinical research methodologies in diagnosis and staging of patients with peritoneal carcinomatosis. Cancer Treat Res. 1996;82:359-74.

25. Yu P, Ye Z, Dai G, Zhang Y, Huang L, Du Y, et al. Neoadjuvant systemic and hyperthermic intraperitoneal chemotherapy combined with cytoreductive surgery for gastric cancer patients with limited peritoneal metastasis: a prospective cohort study. BMC Cancer. 2020;20(1):1108.

26. Canbay E, Mizumoto A, Ichinose M, Ishibashi H, Sako S, Hirano M, et al. Outcome data of patients with peritoneal carcinomatosis from gastric origin treated by a strategy of bidirectional chemotherapy prior to cytoreductive surgery and hyperthermic intraperitoneal chemotherapy in a single specialized center in Japan. Ann Surg Oncol. 2014;21(4):1147-52.

27. Canbay E, Canbay Torun B, Cosarcan K, Altunal C, Gurbuz B, Bilgic C, et al. Surgery with hyperthermic intraperitoneal chemotherapy after response to induction chemotherapy in patients with peritoneal metastasis of gastric cancer. J Gastrointest Oncol. 2021;12(Suppl 1):S47-S56.

28. Manzanedo I, Pereira F, Rihuete Caro C, Perez-Viejo E, Serrano A, Gutierrez Calvo A, et al. Cytoreductive Surgery and Hyperthermic Intraperitoneal Chemotherapy (HIPEC) for Gastric Cancer with Peritoneal Carcinomatosis: Multicenter Study of Spanish Group of Peritoneal Oncologic Surgery (GECOP). Ann Surg Oncol. 2019;26(8):2615-21.

29. Lin T, Chen X, Xu Z, Hu Y, Liu H, Yu J, et al. Laparoscopic cytoreductive surgery and hyperthermic intraperitoneal chemotherapy for gastric cancer with intraoperative detection of limited peritoneal metastasis: a Phase II study of CLASS-05 trial. Gastroenterol Rep (Oxf). 2024;12:goae001.

30. Marano L, Marrelli D, Sammartino P, Biacchi D, Graziosi L, Marino E, et al. Cytoreductive Surgery and Hyperthermic Intraperitoneal Chemotherapy for Gastric Cancer with Synchronous Peritoneal Metastases: Multicenter Study of 'Italian Peritoneal Surface Malignancies Oncoteam-S.I.C.O.'. Ann Surg Oncol. 2021;28(13):9060-70.

31. Merboth F, Garcia S, J VR, Distler M, Welsch T, Weitz J, et al. Comparative Analysis of Postoperative Complications after Cytoreductive Surgery and HIPEC in Gastric Cancer. Oncol Res Treat. 2022;45(1-2):45-53.

32. Rau B, Brandl A, Piso P, Pelz J, Busch P, Demtroder C, et al. Peritoneal metastasis in gastric cancer: results from the German database. Gastric Cancer. 2020;23(1):11-22.

33. Rihuete Caro C, Manzanedo I, Pereira F, Carrion-Alvarez L, Serrano A, Perez-Viejo E. Cytoreductive surgery combined with hyperthermic intraperitoneal chemotherapy (HIPEC) in patients with gastric cancer and peritoneal carcinomatosis. Eur J Surg Oncol. 2018;44(11):1805-10.

34. Torun BC, Sobutay E, Akbulut OE, Saglam S, Yilmaz S, Yonemura Y, et al. Important Predictive Factors for the Prognosis of Patients With Peritoneal Metastasis of Gastric Cancer. Ann Surg Oncol. 2024;31(9):5975-83.

35. Yarema R, Mielko J, Fetsych T, Ohorchak M, Skorzewska M, Rawicz-Pruszynski K, et al. Hyperthermic intraperitoneal chemotherapy (HIPEC) in combined treatment of locally advanced and intraperitonealy disseminated gastric cancer: A retrospective cooperative Central-Eastern European study. Cancer Med. 2019;8(6):2877-85.

36. Yarema R, capital O CcEMC, Hyrya P, Kovalchuk Y, Safiyan V, Karelin I, et al. Gastric cancer with peritoneal metastases: Efficiency of standard treatment methods. World J Gastrointest Oncol. 2020;12(5):569-81.

37. White MG, Kothari A, Ikoma N, Murphy MB, Song S, Ajani J, et al. Factors Associated with Resection and Survival After Laparoscopic HIPEC for Peritoneal Gastric Cancer Metastasis. Ann Surg Oncol. 2020;27(13):4963-9.

38. Reese M, Eichelmann AK, Nowacki TM, Pascher A, Sporn JC. The role of cytoreductive surgery and HIPEC for the treatment of primary and secondary peritoneal malignancies-experience from a tertiary care center in Germany. Langenbecks Arch Surg. 2024;409(1):113.

39. Santullo F, Ferracci F, Abatini C, Halabieh MAE, Lodoli C, D'Annibale G, et al. Gastric cancer with peritoneal metastases: a single center outline and comparison of different surgical and intraperitoneal treatments. Langenbecks Arch Surg. 2023;408(1):437.

40. Bonnot PE, Piessen G, Kepenekian V, Decullier E, Pocard M, Meunier B, et al. Cytoreductive Surgery With or Without Hyperthermic Intraperitoneal Chemotherapy for Gastric Cancer With Peritoneal Metastases (CYTO-CHIP study): A Propensity Score Analysis. J Clin Oncol. 2019;37(23):2028-40.

41. Coccolini F, Catena F, Glehen O, Yonemura Y, Sugarbaker PH, Piso P, et al. Complete versus incomplete cytoreduction in peritoneal carcinosis from gastric cancer, with consideration to PCI cut-off. Systematic review and meta-analysis. Eur J Surg Oncol. 2015;41(7):911-9.

42. Rau B, Lang H, Koenigsrainer A, Gockel I, Rau HG, Seeliger H, et al. Effect of Hyperthermic Intraperitoneal Chemotherapy on Cytoreductive Surgery in Gastric Cancer With Synchronous Peritoneal Metastases: The Phase III GASTRIPEC-I Trial. J Clin Oncol. 2024;42(2):146-56.

43. Ishigami H, Fujiwara Y, Fukushima R, Nashimoto A, Yabusaki H, Imano M, et al. Phase III Trial Comparing Intraperitoneal and Intravenous Paclitaxel Plus S-1 Versus Cisplatin Plus S-1 in Patients With Gastric Cancer With Peritoneal Metastasis: PHOENIX-GC Trial. J Clin Oncol. 2018;36(19):1922-9.

44. Yan C, Yang Z, Shi Z, Lu S, Shi M, Nie M, et al., editors. Intraperitoneal and intravenous paclitaxel plus S-1 versus intravenous paclitaxel plus S-1 in gastric cancer patients with peritoneal metastasis: Results from the multicenter, randomized, phase 3 DRAGON-01 trial. ASCO Gastrointestinal Cancers Symposium; 2025: Journal of Clinical Oncology.

45. Knodler M, Korfer J, Kunzmann V, Trojan J, Daum S, Schenk M, et al. Randomised phase II trial to investigate catumaxomab (anti-EpCAM x anti-CD3) for treatment of peritoneal carcinomatosis in patients with gastric cancer. Br J Cancer. 2018;119(3):296-302.

46. Satoh S, Okabe H, Teramukai S, Hasegawa S, Ozaki N, Ueda S, et al. Phase II trial of combined treatment consisting of preoperative S-1 plus cisplatin followed by gastrectomy and postoperative S-1 for stage IV gastric cancer. Gastric Cancer. 2012;15(1):61-9.

47. Okabe H, Hata H, Hosogi H, Ueda S, Ota S, Kinjo Y, et al. A Phase 2 Study of Induction Chemotherapy Using Docetaxel, Cisplatin, and S-1 for Gastric Cancer with Peritoneal Metastasis (KUGC06). Ann Surg Oncol. 2019;26(6):1779-86.

48. Takeyoshi I, Makita F, Tanahashi Y, Iwazaki S, Ogawa T, Tomizawa N, et al. A phase II study of weekly paclitaxel and doxifluridine combination chemotherapy for advanced/recurrent gastric cancer. Anticancer Res. 2011;31(1):287-91.

49. Sym SJ, Chang HM, Ryu MH, Lee JL, Kim TW, Yook JH, et al. Neoadjuvant docetaxel, capecitabine and cisplatin (DXP) in patients with unresectable locally advanced or metastatic gastric cancer. Ann Surg Oncol. 2010;17(4):1024-32.

50. Yamao T, Shimada Y, Shirao K, Ohtsu A, Ikeda N, Hyodo I, et al. Phase II study of sequential methotrexate and 5-fluorouracil chemotherapy against peritoneally disseminated gastric cancer with malignant ascites: a report from the Gastrointestinal Oncology Study Group of the Japan Clinical Oncology Group, JCOG 9603 Trial. Jpn J Clin Oncol. 2004;34(6):316-22.

51. Guchelaar NAD, Noordman BJ, Welten MW, van Santen MT, de Neijs MJ, Koolen SLW, et al. Systemic Treatment Strategies and Outcomes of Patients With Synchronous Peritoneal Metastases of Gastric Origin: A Nationwide Population-Based Study. J Natl Compr Canc Netw. 2024;22(6):405-12.

52. Hara H, Kadowaki S, Asayama M, Ooki A, Yamada T, Yoshii T, et al. First-line bolus 5-fluorouracil plus leucovorin for peritoneally disseminated gastric cancer with massive ascites or inadequate oral intake. Int J Clin Oncol. 2018;23(2):275-80.

53. Janjigian YY, Shitara K, Moehler M, Garrido M, Salman P, Shen L, et al. First-line nivolumab plus chemotherapy versus chemotherapy alone for advanced gastric, gastro-oesophageal junction, and oesophageal adenocarcinoma (CheckMate 649): a randomised, open-label, phase 3 trial. Lancet. 2021;398(10294):27-40.

54. Shah MA, Shitara K, Ajani JA, Bang YJ, Enzinger P, Ilson D, et al. Zolbetuximab plus CAPOX in CLDN18.2-positive gastric or gastroesophageal junction adenocarcinoma: the randomized, phase 3 GLOW trial. Nat Med. 2023;29(8):2133-41.

55. Janjigian YY, Kawazoe A, Bai Y, Xu J, Lonardi S, Metges JP, et al. Pembrolizumab plus trastuzumab and chemotherapy for HER2-positive gastric or gastro-oesophageal junction adenocarcinoma: interim analyses from the phase 3 KEYNOTE-811 randomised placebo-controlled trial. Lancet. 2023;402(10418):2197-208.

56. Rha SY, Oh DY, Yanez P, Bai Y, Ryu MH, Lee J, et al. Pembrolizumab plus chemotherapy versus placebo plus chemotherapy for HER2-negative advanced gastric cancer (KEYNOTE-859): a multicentre, randomised, double-blind, phase 3 trial. Lancet Oncol. 2023;24(11):1181-95.

57. Shitara K, Lordick F, Bang YJ, Enzinger P, Ilson D, Shah MA, et al. Zolbetuximab plus mFOLFOX6 in patients with CLDN18.2-positive, HER2-negative, untreated, locally advanced unresectable or metastatic gastric or gastro-oesophageal junction adenocarcinoma (SPOTLIGHT): a multicentre, randomised, double-blind, phase 3 trial. Lancet. 2023;401(10389):1655-68.

58. Arai H, Kawahira M, Yasui H, Masuishi T, Muro K, Nakajima TE. Second-line chemotherapy using taxane in patients with advanced gastric cancer who presented with severe peritoneal metastasis: a multicenter retrospective study. Int J Clin Oncol. 2021;26(2):355-63.

59. Arai H, Inoue E, Yamaguchi K, Boku N, Hara H, Nishina T, et al. Clinical implications of using both fluoropyrimidine and paclitaxel in patients with severe peritoneal metastasis of gastric cancer: A post hoc study of JCOG1108/WJOG7312G. Cancer Med. 2021;10(21):7673-82.

60. Nakajima TE, Yamaguchi K, Boku N, Hyodo I, Mizusawa J, Hara H, et al. Randomized phase II/III study of 5-fluorouracil/l-leucovorin versus 5-fluorouracil/l-leucovorin plus paclitaxel administered to patients with severe peritoneal metastases of gastric cancer (JCOG1108/WJOG7312G). Gastric Cancer. 2020;23(4):677-88.

61. Fuchs CS, Shitara K, Di Bartolomeo M, Lonardi S, Al-Batran SE, Van Cutsem E, et al. Ramucirumab with cisplatin and fluoropyrimidine as first-line therapy in patients with metastatic gastric or junctional adenocarcinoma (RAINFALL): a double-blind, randomised, placebo-controlled, phase 3 trial. Lancet Oncol. 2019;20(3):420-35.

62. Takashima A, Shitara K, Fujitani K, Koeda K, Hara H, Nakayama N, et al. Peritoneal metastasis as a predictive factor for nab-paclitaxel in patients with pretreated advanced gastric cancer: an exploratory analysis of the phase III ABSOLUTE trial. Gastric Cancer. 2019;22(1):155-63.

63. Shitara K, Takashima A, Fujitani K, Koeda K, Hara H, Nakayama N, et al. Nab-paclitaxel versus solvent-based paclitaxel in patients with previously treated advanced gastric cancer (ABSOLUTE): an open-label, randomised, non-inferiority, phase 3 trial. Lancet Gastroenterol Hepatol. 2017;2(4):277-87.

64. Nishina T, Boku N, Gotoh M, Shimada Y, Hamamoto Y, Yasui H, et al. Randomized phase II study of second-line chemotherapy with the best available 5-fluorouracil regimen versus weekly administration of paclitaxel in far advanced gastric cancer with severe peritoneal metastases refractory to 5-fluorouracil-containing regimens (JCOG0407). Gastric Cancer. 2016;19(3):902-10.

65. Hironaka S, Ueda S, Yasui H, Nishina T, Tsuda M, Tsumura T, et al. Randomized, open-label, phase III study comparing irinotecan with paclitaxel in patients with advanced gastric cancer without severe peritoneal metastasis after failure of prior combination chemotherapy using fluoropyrimidine plus platinum: WJOG 4007 trial. J Clin Oncol. 2013;31(35):4438-44.

66. Takashima A, Boku N, Kato K, Nakamura K, Mizusawa J, Fukuda H, et al. Survival prolongation after treatment failure of first-line chemotherapy in patients with advanced gastric cancer: combined analysis of the Japan Clinical Oncology group trials JCOG9205 and JCOG9912. Gastric Cancer. 2014;17(3):522-8.

67. Qiu MZ, Oh DY, Kato K, Arkenau T, Tabernero J, Correa MC, et al. Tislelizumab plus chemotherapy versus placebo plus chemotherapy as first line treatment for advanced gastric or gastro-oesophageal junction adenocarcinoma: RATIONALE-305 randomised, double blind, phase 3 trial. BMJ. 2024;385:e078876.

68. Eveno C, Kepenekian V, Brigand C, De Franco V, Sgarbura O, Fontanier S, et al. PIPAC EstoK01: randomized phase II study on Doxorubicin/Cisplatin Pressurized Intra Peritoneal Aerosol Chemotherapy in gastric peritoneal metastasis: postoperative and oncological outcomes. EJSO. 2024;50(2):107377.

69. Bin Y, Lan D, Bao W, Yang H, Zhou S, Huang F, et al. SOX combined with intraperitoneal perfusion of docetaxel compared with DOS regimen in the first-line therapy for advanced gastric cancer with malignant ascites: a prospective observation. Trials. 2022;23(1):211.

70. Ba MC, Long H, Zhang XL, Gong YF, Tang YQ, Wu YB, et al. Laparoscopic Hyperthermic Intraperitoneal Perfusion Chemotherapy for Patients with Malignant Ascites Secondary to Unresectable Gastric Cancer. J Laparoendosc Adv Surg Tech A. 2016;26(1):32-9.

71. Ishigami H, Yamaguchi H, Yamashita H, Asakage M, Kitayama J. Surgery after intraperitoneal and systemic chemotherapy for gastric cancer with peritoneal metastasis or positive peritoneal cytology findings. Gastric Cancer. 2017;20(Suppl 1):128-34.

72. Wang JB, Liu ZY, Huang XB, Chen QY, Zhong Q, Li P, et al. Implications for restaging in gastric cancer with peritoneal metastasis based on the 15th Japanese Classification of Gastric Carcinoma: An analysis from a comprehensive center. Eur J Surg Oncol. 2020;46(7):1269-76.

73. Glehen O, Gilly FN, Arvieux C, Cotte E, Boutitie F, Mansvelt B, et al. Peritoneal carcinomatosis from gastric cancer: a multi-institutional study of 159 patients treated by cytoreductive surgery combined with perioperative intraperitoneal chemotherapy. Ann Surg Oncol. 2010;17(9):2370-7.

74. Yang XJ, Huang CQ, Suo T, Mei LJ, Yang GL, Cheng FL, et al. Cytoreductive surgery and hyperthermic intraperitoneal chemotherapy improves survival of patients with peritoneal carcinomatosis from gastric cancer: final results of a phase III randomized clinical trial. Ann Surg Oncol. 2011;18(6):1575-81.

75. Koemans WJ, van der Kaaij RT, Wassenaar ECE, Boerma D, Boot H, Sikorska K, et al. Tumor characteristics and clinical outcome of peritoneal metastasis of gastric origin treated with a hyperthermic intraperitoneal chemotherapy procedure in the PERISCOPE I trial. J Surg Oncol. 2021;123(4):904-10.

76. Seshadri RA, Hemanth Raj E. Diagnostic Laparoscopy in the Pre-operative Assessment of Patients Undergoing Cytoreductive Surgery and HIPEC for Peritoneal Surface Malignancies. Indian J Surg Oncol. 2016;7(2):230-5.

77. De Andrade JP, Mezhir JJ. The critical role of peritoneal cytology in the staging of gastric cancer: an evidence-based review. J Surg Oncol. 2014;110(3):291-7.

78. Solass W, Sempoux C, Detlefsen S, Carr NJ, Bibeau F. Peritoneal sampling and histological assessment of therapeutic response in peritoneal metastasis: proposal of the Peritoneal Regression Grading Score (PRGS). Pleura Peritoneum. 2016;1(2):99-107.

79. Nakamura M, Ojima T, Nakamori M, Katsuda M, Tsuji T, Hayata K, et al. Conversion Surgery for Gastric Cancer with Peritoneal Metastasis Based on the Diagnosis of Second-Look Staging Laparoscopy. J Gastrointest Surg. 2019;23(9):1758-66.

80. Shinkai M, Imano M, Kohda M, Nakanishi T, Hiraki Y, Hagi T, et al. Efficacy of palliative surgery for gastric cancer patients with peritoneal metastasis who still have residual peritoneal dissemination after chemotherapy. Langenbecks Arch Surg. 2023;408(1):291.

81. Solaini L, Ministrini S, Bencivenga M, D'Ignazio A, Marino E, Cipollari C, et al. Conversion gastrectomy for stage IV unresectable gastric cancer: a GIRCG retrospective cohort study. Gastric Cancer. 2019;22(6):1285-93.

82. Shinkai M, Imano M, Hiraki Y, Momose K, Kato H, Shiraishi O, et al. Efficacy of conversion surgery after a single intraperitoneal administration of paclitaxel and systemic chemotherapy for gastric cancer with peritoneal metastasis. Langenbecks Arch Surg. 2022;407(3):975-83.

83. Kim SW. The result of conversion surgery in gastric cancer patients with peritoneal seeding. J Gastric Cancer. 2014;14(4):266-70.

84. Valletti M, Eshmuminov D, Gnecco N, Gutschow CA, Schneider PM, Lehmann K. Gastric cancer with positive peritoneal cytology: survival benefit after induction chemotherapy and conversion to negative peritoneal cytology. World J Surg Oncol. 2021;19(1):245.

85. Bencivenga M, Ministrini S, Morgagni P, Mura G, Marrelli D, Milandri C, et al. Oligometastatic Gastric Cancer: Clinical Data from the Meta-Gastro Prospective Register of the Italian Research Group on Gastric Cancer. Cancers (Basel). 2023;16(1).

86. Arigami T, Matsushita D, Okubo K, Sasaki K, Noda M, Kita Y, et al. Clinical Significance of Conversion Surgery for Gastric Cancer with Peritoneal Dissemination: A Retrospective Study. Oncology. 2020;98(11):798-806.

87. Yu P, Ding G, Huang X, Wang C, Fang J, Huang L, et al. Genomic and immune microenvironment features influencing chemoimmunotherapy response in gastric cancer with peritoneal metastasis: a retrospective cohort study. Int J Surg. 2024;110(6):3504-17.

88. Yoshida K, Yasufuku I, Terashima M, Young Rha S, Moon Bae J, Li G, et al. International Retrospective Cohort Study of Conversion Therapy for Stage IV Gastric Cancer 1 (CONVO-GC-1). Ann Gastroenterol Surg. 2022;6(2):227-40.

89. Kano Y, Ichikawa H, Hanyu T, Muneoka Y, Ishikawa T, Aizawa M, et al. Conversion surgery for stage IV gastric cancer: a multicenter retrospective study. BMC Surg. 2022;22(1):428.

90. Chan DY, Syn NL, Yap R, Phua JN, Soh TI, Chee CE, et al. Conversion Surgery Post-Intraperitoneal Paclitaxel and Systemic Chemotherapy for Gastric Cancer Carcinomatosis Peritonei. Are We Ready? J Gastrointest Surg. 2017;21(3):425-33.

91. Chia DKA, Sundar R, Kim G, Ang JJ, Lum JHY, Nga ME, et al. Outcomes of a Phase II Study of Intraperitoneal Paclitaxel plus Systemic Capecitabine and Oxaliplatin (XELOX) for Gastric Cancer with Peritoneal Metastases. Ann Surg Oncol. 2022;29(13):8597-605.

92. Shi M, Yang Z, Lu S, Liu W, Ni Z, Yao X, et al. Oxaliplatin plus S-1 with intraperitoneal paclitaxel for the treatment of Chinese advanced gastric cancer with peritoneal metastases. BMC Cancer. 2021;21(1):1344.

93. Tu L, Zhang W, Ni L, Xu Z, Yang K, Gou H, et al. Study of SOX combined with intraperitoneal high-dose paclitaxel in gastric cancer with synchronous peritoneal metastasis: A phase II single-arm clinical trial. Cancer Med. 2023;12(4):4161-9.

94. Yang Z, Lu S, Shi M, Yuan H, Wang Z, Ni Z, et al. Oncological outcomes of conversion therapy in gastric cancer patients with peritoneal metastasis: a large-scale retrospective cohort study. Gastric Cancer. 2024;27(2):387-99.

95. Yamaguchi H, Satoh Y, Ishigami H, Kurihara M, Yatomi Y, Kitayama J. Peritoneal Lavage CEA mRNA Levels Predict Conversion Gastrectomy Outcomes after Induction Chemotherapy with Intraperitoneal Paclitaxel in Gastric Cancer Patients with Peritoneal Metastasis. Ann Surg Oncol. 2017;24(11):3345-52.

96. Yang ZY, Yuan F, Lu S, Xu W, Wu JW, Xi WQ, et al. Efficacy and Safety of Conversion Therapy by Intraperitoneal and Intravenous Paclitaxel Plus Oral S-1 in Gastric Cancer Patients With Peritoneal Metastasis: A Prospective Phase II Study. Front Oncol. 2022;12:905922.

97. Zhang X, Huang H, Yang D, Wang P, Huang X, Hu Z, et al. Neoadjuvant Intraperitoneal and Systemic Chemotherapy Versus Neoadjuvant Systemic Chemotherapy With Docetaxel, Oxaliplatin, and S-1 for Gastric Cancer With Peritoneal Metastasis: A Propensity Score Matched Analysis. Technol Cancer Res Treat. 2021;20:15330338211036310.

98. Morgagni P, Solaini L, Saragoni L, Monti M, Valgiusti M, Vittimberga G, et al. Conversion Surgery in Gastric Cancer Carcinomatosis. Front Oncol. 2022;12:852559.

99. Lee TY, Liao GS, Fan HL, Hsieh CB, Chen TW, Chan DC. Conversion Surgery for Patients with Advanced Gastric Cancer with Peritoneal Carcinomatosis. J Oncol. 2021;2021:5459432.

100. Yonemura Y, Prabhu A, Sako S, Ishibashi H, Mizumoto A, Takao N, et al. Long Term Survival after Cytoreductive Surgery Combined with Perioperative Chemotherapy in Gastric Cancer Patients with Peritoneal Metastasis. Cancers (Basel). 2020;12(1).

101. Guo J, Deng Z, Jin L, Yin S, Xiong Z, Wang C, et al. Prognostic value of hyperthermic intraperitoneal chemotherapy in gastric cancer with synchronous peritoneal metastases: a real-world retrospective study. J Cancer Res Clin Oncol. 2023;149(20):17881-96.

102. Kobialka S, Sedlak K, Pelc Z, Mlak R, Endo Y, Bogacz P, et al. Hyperthermic Intraperitoneal Chemotherapy (HIPEC), Oncological Outcomes and Long-Term Survival among Patients with Gastric Cancer and Limited Peritoneal Disease Progression after Neoadjuvant Chemotherapy. J Clin Med. 2023;13(1).

103. Ji ZH, Yu Y, Liu G, Zhang YB, An SL, Li B, et al. Peritoneal cancer index (PCI) based patient selecting strategy for complete cytoreductive surgery plus hyperthermic intraperitoneal chemotherapy in gastric cancer with peritoneal metastasis: A single-center retrospective analysis of 125 patients. Eur J Surg Oncol. 2021;47(6):1411-9.

104. Chia CS, You B, Decullier E, Vaudoyer D, Lorimier G, Abboud K, et al. Patients with Peritoneal Carcinomatosis from Gastric Cancer Treated with Cytoreductive Surgery and Hyperthermic Intraperitoneal Chemotherapy: Is Cure a Possibility? Ann Surg Oncol. 2016;23(6):1971-9.

105. Mielko J, Rawicz-Pruszynski K, Skorzewska M, Cisel B, Pikula A, Kwietniewska M, et al. Conversion Surgery with HIPEC for Peritoneal Oligometastatic Gastric Cancer. Cancers (Basel). 2019;11(11).

106. Green BL, Blumenthaler AN, Gamble LA, McDonald JD, Robinson K, Connolly M, et al. Cytoreduction and HIPEC for Gastric Carcinomatosis: Multi-institutional Analysis of Two Phase II Clinical Trials. Ann Surg Oncol. 2023;30(3):1852-60.

107. Cho M, Kim HS, Jung M, Hyung WJ. Perioperative intraperitoneal plus systemic chemotherapy and cytoreductive surgery with hyperthermic intraperitoneal chemotherapy for gastric cancer: phase Ib/II single-arm prospective study. J Gastrointest Surg. 2024;28(7):1095-103.

108. Buckarma E, Thiels CA, Jin Z, Grotz TE. Cytoreduction and Hyperthermic Intraperitoneal Paclitaxel and Cisplatin for Gastric Cancer with Peritoneal Metastasis. Ann Surg Oncol. 2024;31(1):622-9.

109. Yu HH, Yonemura Y, Ng HJ, Lee MC, Su BC, Hsieh MC. Benefit of Neoadjuvant Laparoscopic Hyperthermic Intraperitoneal Chemotherapy and Bidirectional Chemotherapy for Patients with Gastric Cancer with Peritoneal Carcinomatosis Considering Cytoreductive Surgery. Cancers (Basel). 2023;15(13).

110. Kim DW, Park DG, Song S, Jee YS. Cytoreductive Surgery and Hyperthermic Intraperitoneal Chemotherapy as Treatment Options for Peritoneal Metastasis of Advanced Gastric Cancer. J Gastric Cancer. 2018;18(3):296-304.

111. Rau B, Brandl A, Thuss-Patience P, Bergner F, Raue W, Arnold A, et al. The efficacy of treatment options for patients with gastric cancer and peritoneal metastasis. Gastric Cancer. 2019;22(6):1226-37.

112. Al-Batran S, Lorenzen S, Riera J, Caca K, Mueller CL, Stange DE, et al. Effect of chemotherapy/targeted therapy alone vs. chemotherapy/targeted therapy followed by radical surgical resection on survival and quality of life in patients with limited metastatic adenocarcinoma of the stomach or esophagogastric junction: the IKF-575/RENAISSANCE phase III trial. 2024 ASCO Annual Meeting II. Journal of Clinical Oncology. 2024;42.

113. Sanders JJ, Temin S, Ghoshal A, Alesi ER, Ali ZV, Chauhan C, et al. Palliative Care for Patients With Cancer: ASCO Guideline Update. J Clin Oncol. 2024;42(19):2336-57.

114. Dans M, Kutner JS, Agarwal R, Baker JN, Bauman JR, Beck AC, et al. NCCN Guidelines(R) Insights: Palliative Care, Version 2.2021. J Natl Compr Canc Netw. 2021;19(7):780-8.

115. Fan B, Bu Z, Zhang J, Zong X, Ji X, Fu T, et al. Phase II trial of prophylactic hyperthermic intraperitoneal chemotherapy in patients with locally advanced gastric cancer after curative surgery. BMC Cancer. 2021;21(1):216.

116. Koga S, Hamazoe R, Maeta M, Shimizu N, Murakami A, Wakatsuki T. Prophylactic therapy for peritoneal recurrence of gastric cancer by continuous hyperthermic peritoneal perfusion with mitomycin C. Cancer. 1988;61(2):232-7.

117. Hamazoe R, Maeta M, Kaibara N. Intraperitoneal thermochemotherapy for prevention of peritoneal recurrence of gastric cancer. Final results of a randomized controlled study. Cancer. 1994;73(8):2048-52.

118. Sautner T, Hofbauer F, Depisch D, Schiessel R, Jakesz R. Adjuvant intraperitoneal cisplatin chemotherapy does not improve long-term survival after surgery for advanced gastric cancer. J Clin Oncol. 1994;12(5):970-4.

119. Fujimura T, Yonemura Y, Muraoka K, Takamura H, Hirono Y, Sahara H, et al. Continuous hyperthermic peritoneal perfusion for the prevention of peritoneal recurrence of gastric cancer: randomized controlled study. World J Surg. 1994;18(1):150-5.

120. Fujimoto S, Takahashi M, Mutou T, Kobayashi K, Toyosawa T. Successful intraperitoneal hyperthermic chemoperfusion for the prevention of postoperative peritoneal recurrence in patients with advanced gastric carcinoma. Cancer. 1999;85(3):529-34.

121. Yu W, Whang I, Chung HY, Averbach A, Sugarbaker PH. Indications for early postoperative intraperitoneal chemotherapy of advanced gastric cancer: results of a prospective randomized trial. World J Surg. 2001;25(8):985-90.

122. Cui HB, Ge HE, Bai XY, Zhang W, Zhang YY, Wang J, et al. Effect of neoadjuvant chemotherapy combined with hyperthermic intraperitoneal perfusion chemotherapy on advanced gastric cancer. Exp Ther Med. 2014;7(5):1083-8.

123. Reutovich MY, Krasko OV, Sukonko OG. Hyperthermic intraperitoneal chemotherapy in serosa-invasive gastric cancer patients. Eur J Surg Oncol. 2019;45(12):2405-11.

124. Liu L, Sun L, Zhang N, Liao CG, Su H, Min J, et al. A novel method of bedside hyperthermic intraperitoneal chemotherapy as adjuvant therapy for stage-III gastric cancer. Int J Hyperthermia. 2022;39(1):239-45.

125. Lee TY, Hsu CH, Fan HL, Liao GS, Chen TW, Chan DC. Prophylactic hyperthermic intraperitoneal chemotherapy for patients with clinical T4 gastric cancer. Eur J Surg Oncol. 2022;48(9):1972-9.

126. Allievi N, Bianco F, Pisano M, Montori G, Fugazzola P, Coccolini F, et al. Hyperthermic intraperitoneal chemotherapy (HIPEC) as adjuvant and therapeutic options for patients with advanced gastric cancer at high risk of recurrence or established peritoneal metastases: a single-centre experience. Updates Surg. 2023;75(1):159-67.

127. Xie TY, Wu D, Li S, Qiu ZY, Song QY, Guan D, et al. Role of prophylactic hyperthermic intraperitoneal chemotherapy in patients with locally advanced gastric cancer. World J Gastrointest Oncol. 2020;12(7):782-90.

128. Zhu L, Xu Z, Wu Y, Liu P, Qian J, Yu S, et al. Prophylactic chemotherapeutic hyperthermic intraperitoneal perfusion reduces peritoneal metastasis in gastric cancer: a retrospective clinical study. BMC Cancer. 2020;20(1):827.

129. Coccolini F, Celotti A, Ceresoli M, Montori G, Marini M, Catena F, et al. Hyperthermic intraperitoneal chemotherapy (HIPEC) and neoadjuvant chemotherapy as prophylaxis of peritoneal carcinosis from advanced gastric cancer-effects on overall and disease free survival. J Gastrointest Oncol. 2016;7(4):523-9.

130. Miyashiro I, Furukawa H, Sasako M, Yamamoto S, Nashimoto A, Nakajima T, et al. Randomized clinical trial of adjuvant chemotherapy with intraperitoneal and intravenous cisplatin followed by oral fluorouracil (UFT) in serosa-positive gastric cancer versus curative resection alone: final results of the Japan Clinical Oncology Group trial JCOG9206-2. Gastric Cancer. 2011;14(3):212-8.

131. Rosen HR, Jatzko G, Repse S, Potrc S, Neudorfer H, Sandbichler P, et al. Adjuvant intraperitoneal chemotherapy with carbon-adsorbed mitomycin in patients with gastric cancer: results of a randomized multicenter trial of the Austrian Working Group for Surgical Oncology. J Clin Oncol. 1998;16(8):2733-8.

132. Kang YK, Yook JH, Chang HM, Ryu MH, Yoo C, Zang DY, et al. Enhanced efficacy of postoperative adjuvant chemotherapy in advanced gastric cancer: results from a phase 3 randomized trial (AMC0101). Cancer Chemother Pharmacol. 2014;73(1):139-49.

133. Shimoyama S, Shimizu N, Kaminishi M. Type-oriented intraoperative and adjuvant chemotherapy and survival after curative resection of advanced gastric cancer. World J Surg. 1999;23(3):284-91; discussion 91-2.

134. Graversen M, Rouvelas I, Ainsworth AP, Bjarnesen AP, Detlefsen S, Ellebaek SB, et al. Feasibility and Safety of Laparoscopic D2 Gastrectomy in Combination with Pressurized Intraperitoneal Aerosol Chemotherapy (PIPAC) in Patients with Gastric Cancer at High Risk of Recurrence-The PIPAC-OPC4 Study. Ann Surg Oncol. 2023;30(7):4433-41.

135. Jiang Y, Liang X, Wang W, Chen C, Yuan Q, Zhang X, et al. Noninvasive Prediction of Occult Peritoneal Metastasis in Gastric Cancer Using Deep Learning. JAMA Netw Open. 2021;4(1):e2032269.

136. Huang Z, Liu D, Chen X, He D, Yu P, Liu B, et al. Deep Convolutional Neural Network Based on Computed Tomography Images for the Preoperative Diagnosis of Occult Peritoneal Metastasis in Advanced Gastric Cancer. Front Oncol. 2020;10:601869.

137. Dong D, Tang L, Li ZY, Fang MJ, Gao JB, Shan XH, et al. Development and validation of an individualized nomogram to identify occult peritoneal metastasis in patients with advanced gastric cancer. Ann Oncol. 2019;30(3):431-8.

138. Kim HY, Kim YH, Yun G, Chang W, Lee YJ, Kim B. Could texture features from preoperative CT image be used for predicting occult peritoneal carcinomatosis in patients with advanced gastric cancer? PLoS One. 2018;13(3):e0194755.

139. Huang W, Zhou K, Jiang Y, Chen C, Yuan Q, Han Z, et al. Radiomics Nomogram for Prediction of Peritoneal Metastasis in Patients With Gastric Cancer. Front Oncol. 2020;10:1416.

140. Jiang Y, Zhang Z, Yuan Q, Wang W, Wang H, Li T, et al. Predicting peritoneal recurrence and disease-free survival from CT images in gastric cancer with multitask deep learning: a retrospective study. Lancet Digit Health. 2022;4(5):e340-e50.

141. Hasegawa H, Fujitani K, Nakazuru S, Hirao M, Yamamoto K, Mita E, et al. Optimal treatment change criteria for advanced gastric cancer with non-measurable peritoneal metastasis: symptom/tumor marker-based versus CT-based. Anticancer Res. 2014;34(9):5169-74.

142. Zhou C, Wang Y, Ji MH, Tong J, Yang JJ, Xia H. Predicting Peritoneal Metastasis of Gastric Cancer Patients Based on Machine Learning. Cancer Control. 2020;27(1):1073274820968900.

143. Ruan D, Zhao L, Cai J, Xu W, Sun L, Li J, et al. Evaluation of FAPI PET imaging in gastric cancer: a systematic review and meta-analysis. Theranostics. 2023;13(13):4694-710.

144. Pang Y, Zhao L, Luo Z, Hao B, Wu H, Lin Q, et al. Comparison of (68)Ga-FAPI and (18)F-FDG Uptake in Gastric, Duodenal, and Colorectal Cancers. Radiology. 2021;298(2):393-402.

145. De Vuysere S, Vandecaveye V, De Bruecker Y, Carton S, Vermeiren K, Tollens T, et al. Accuracy of whole-body diffusion-weighted MRI (WB-DWI/MRI) in diagnosis, staging and follow-up of gastric cancer, in comparison to CT: a pilot study. BMC Med Imaging. 2021;21(1):18.

146. Lin CN, Huang WS, Huang TH, Chen CY, Huang CY, Wang TY, et al. Adding Value of MRI over CT in Predicting Peritoneal Cancer Index and Completeness of Cytoreduction. Diagnostics (Basel). 2021;11(4).

147. Yanagihara K, Takigahira M, Takeshita F, Komatsu T, Nishio K, Hasegawa F, et al. A photon counting technique for quantitatively evaluating progression of peritoneal tumor dissemination. Cancer Res. 2006;66(15):7532-9.

148. Li Z, Wang J, Wang Z, Xu Y. Towards an optimal model for gastric cancer peritoneal metastasis: current challenges and future directions. EBioMedicine. 2023;92:104601.

149. Shimura T, Toden S, Kandimalla R, Toiyama Y, Okugawa Y, Kanda M, et al. Genomewide Expression Profiling Identifies a Novel miRNA-based Signature for the Detection of Peritoneal Metastasis in Patients With Gastric Cancer. Ann Surg. 2021;274(5):e425-e34.

150. Ohzawa H, Kumagai Y, Yamaguchi H, Miyato H, Sakuma Y, Horie H, et al. Exosomal microRNA in peritoneal fluid as a biomarker of peritoneal metastases from gastric cancer. Ann Gastroenterol Surg. 2020;4(1):84-93.

151. van der Sluis K, van Sandick JW, Vollebergh MA, van Dieren JM, Hugen N, Hartemink KJ, et al. Improving diagnostic accuracy of identifying gastric cancer patients with peritoneal metastases: tumor-guided cell-free DNA analysis of peritoneal fluid. Oncogene. 2024;43(24):1877-82.

152. Gwee YX, Chia DKA, So J, Ceelen W, Yong WP, Tan P, et al. Integration of Genomic Biology Into Therapeutic Strategies of Gastric Cancer Peritoneal Metastasis. J Clin Oncol. 2022;40(24):2830.

153. Allan Z, Witts S, Wong DJ, Lee MM, Tie J, Tebbutt NC, et al. Peritoneal Tumor DNA as a Prognostic Biomarker in Gastric Cancer: A Systematic Review and Meta-Analysis. JCO Precis Oncol. 2024;8:e2300546.

154. Jin J, Son M, Kim H, Kim H, Kong SH, Kim HK, et al. Comparative proteomic analysis of human malignant ascitic fluids for the development of gastric cancer biomarkers. Clin Biochem. 2018;56:55-61.

155. Ueda A, Yuki S, Ando T, Hosokawa A, Nakada N, Kito Y, et al. CA125 Kinetics as a Potential Biomarker for Peritoneal Metastasis Progression following Taxane-Plus-Ramucirumab Administration in Patients with Advanced Gastric Cancer. Cancers (Basel). 2024;16(5).

156. Emoto S, Ishigami H, Yamashita H, Yamaguchi H, Kaisaki S, Kitayama J. Clinical significance of CA125 and CA72-4 in gastric cancer with peritoneal dissemination. Gastric Cancer. 2012;15(2):154-61.

157. Xu X, Dang Z, Zhang J, Feng Y, Wei Z. The miRNA, miR-125b, Inhibited Invasion and Metastasis of Gastric-Cancer Cells by Triggering the STAT3 Signaling Pathway. Cancer Manag Res. 2020;12:8569-80.

158. Gao P, Wang S, Jing F, Zhan J, Wang Y. microRNA-203 suppresses invasion of gastric cancer cells by targeting ERK1/2/Slug/ E-cadherin signaling. Cancer Biomark. 2017;19(1):11-20.

159. Zheng J, Ge P, Liu X, Wei J, Wu G, Li X. MiR-136 inhibits gastric cancer-specific peritoneal metastasis by targeting HOXC10. Tumour Biol. 2017;39(6):1010428317706207.

160. Sun F, Yu M, Yu J, Liu Z, Zhou X, Liu Y, et al. miR-338-3p functions as a tumor suppressor in gastric cancer by targeting PTP1B. Cell Death Dis. 2018;9(5):522.

161. Li Z, Zhang G, Li D, Jie Z, Chen H, Xiong J, et al. Methylation-associated silencing of miR-495 inhibit the migration and invasion of human gastric cancer cells by directly targeting PRL-3. Biochem Biophys Res Commun. 2015;456(1):344-50.

162. Berretta M, Fisichella R, Borsatti E, Lleshi A, Ioffredo S, Meneguzzo N, et al. Feasibility of intraperitoneal Trastuzumab treatment in a patient with peritoneal carcinomatosis from gastric cancer. Eur Rev Med Pharmacol Sci. 2014;18(5):689-92.

163. Qi C, Wu S, Kim I-H, Cai S, Wang J, Kim ST, et al. Global multi-center phase I trial of the intraperitoneal infusion of anti-EpCAM x anti-CD3 bispecific antibody catumaxomab for advanced gastric carcinoma with peritoneal metastasis. ASCO 2022 Online 40(16 Supplement 1). 2022.

164. Kumagai Y, Futoh Y, Miyato H, Ohzawa H, Yamaguchi H, Saito S, et al. Effect of Systemic or Intraperitoneal Administration of Anti-PD-1 Antibody for Peritoneal Metastases from Gastric Cancer. In Vivo. 2022;36(3):1126-35.

165. Tabuchi M, Kikuchi S, Tazawa H, Okura T, Ogawa T, Mitsui E, et al. Functional remodeling of intraperitoneal macrophages by oncolytic adenovirus restores anti-tumor immunity for peritoneal metastasis of gastric cancer. Mol Ther Oncol. 2024;32(2):200806.

166. Zhang B, Huang J, Tang J, Hu S, Luo S, Luo Z, et al. Intratumoral OH2, an oncolytic herpes simplex virus 2, in patients with advanced solid tumors: a multicenter, phase I/II clinical trial. J Immunother Cancer. 2021;9(4).

167. Li HK, Morokoshi Y, Nagatsu K, Kamada T, Hasegawa S. Locoregional therapy with alpha-emitting trastuzumab against peritoneal metastasis of human epidermal growth factor receptor 2-positive gastric cancer in mice. Cancer Sci. 2017;108(8):1648-56.

168. Pinto A, Pocard M. Photodynamic therapy and photothermal therapy for the treatment of peritoneal metastasis: a systematic review. Pleura Peritoneum. 2018;3(4):20180124.

169. Larsen SG, Graf W, Mariathasan AB, Sorensen O, Spasojevic M, Goscinski MA, et al. First experience with (224)Radium-labeled microparticles (Radspherin(R)) after CRS-HIPEC for peritoneal metastasis in colorectal cancer (a phase 1 study). Front Med (Lausanne). 2023;10:1070362.

170. Thorgersen EB, Asvall J, Schjalm C, McAdam KE, Bruland OS, Larsen SG, et al. Effect of Intraperitoneal (224)Radium-Labelled Microparticles on Compartmentalized Inflammation After Cytoreductive Surgery and Hypertherm Intraperitoneal Chemotherapy. Technol Cancer Res Treat. 2023;22:15330338231192902.

171. Larsen SG, Graf W, Larsen RH, Revheim ME, Mariathasan AM, Sorensen O, et al. Eighteen-Months Safety and Efficacy Following Intraperitoneal Treatment With (224)Radium-Labeled Microparticles After CRS-HIPEC in Patients With Peritoneal Metastasis From Colorectal Cancer. J Surg Oncol. 2024;130(6):1395-402.

172. Willaert W, Van de Sande L, Van Daele E, Van De Putte D, Van Nieuwenhove Y, Pattyn P, et al. Safety and preliminary efficacy of electrostatic precipitation during pressurized intraperitoneal aerosol chemotherapy (PIPAC) for unresectable carcinomatosis. Eur J Surg Oncol. 2019;45(12):2302-9.

173. Kryh-Jensen CG, Fristrup CW, Ainsworth AP, Detlefsen S, Mortensen MB, Pfeiffer P, et al. What is long-term survival in patients with peritoneal metastasis from gastric, pancreatic, or colorectal cancer? A study of patients treated with systemic chemotherapy and pressurized intraperitoneal aerosol chemotherapy (PIPAC). Pleura Peritoneum. 2023;8(4):147-55.

174. Gangannapalle M, Shahnoor H, Sattar L, Nagi TK, Al-Tekreeti M, Khan MW, et al. Nanoparticle Albumin‑Bound Paclitaxel and Solvent-Based Paclitaxel as Chemotherapy Options for Patients With Advanced Gastric Cancer: A Systematic Review and Meta-Analysis. Cureus. 2023;15(7):e41711.

175. Chen ZX, Li J, Liu WB, Zhang SR, Sun H. Elemene-containing hyperthermic intraperitoneal chemotherapy combined with chemotherapy for elderly patients with peritoneal metastatic advanced gastric cancer. World J Clin Cases. 2022;10(5):1498-507.

176. Jiang WG, Jia Y, Ye L, Ji K, Gao Y, Wei C, et al. Abstract 4034: The anti-peritoneal metastasis properties of Yangzheng Xiaoji, the potential role of hyaluronan and CD44 Cancer Res (2014) 74 (19_Supplement): 4034. 2014.

177. Ji K, Jia Y, Gao Y, Wei C, Wu Y, Topley N, et al. The biological and in vivo impact of Yangzheng Xiaoji extract on the interaction between tumour and peritoneal mesothelial cells and peritoneal metastases of gastrointestinal tumours. European Journal of Cancer Volume 51, Supplement 3, September 2015, Page S462. 2015.
